# Supplementary material for: Cyclic Dipeptides: The Biological and Structural Landscape with Special Focus on the Anti-Cancer Proline-Based Scaffold
Source: Biomolecules. 2021 Oct 14;11(10):1515. doi: 10.3390/biom11101515 (PMC8533947; doi:10.3390/biom11101515)
Supplement: Supplementary file 1 [file biomolecules-11-01515-s001.zip › biomolecules-1406865-supplementary.pdf]

Biomolecules
Special issue: Selected Papers from the 1st International Electronic Conference on Biomolecules

SUPLEMENTARY MATERIALS

Diketopiperazines: the biological and structural landscape with special focus on the anti-cancer proline-based DKP scaffold

Joanna Bojarska,* Adam Mieczkowski, Zyta Ziora, Mariusz Skwarczynski, Istvan Toth, Ahmed O. Shalash, Keykavous Parang, Shaima A. El-Mowafi, Eman H. M. Mohammed, Sherif Elnagdy, Maha AlKhazindar, Wojciech M. Wolf

* Correspondence: joanna.bojarska@p.lodz.pl

The overwiew of the structural databases.

The Research Collaboratory for Structural Bioinformatics Protein Data Bank

The survey of the Research Collaboratory for Structural Bioinformatics Protein Data Bank (RCSB PDB) revealed 42 bio-complexes related to DKP moiety, including proline-based one, signed by reference codes: 6SSG, 6SSF, 6SSE, 6SSD, 6F0B, 6F0C, 41CT, 41Q7, 41PS, 41PW, 41Q9, 5YL4, 6EZ3, 5MLQ, 5OCD, 5MLP, 4Q24, 4E0T, 4E0U, 3S7T, 3OQJ, 3OQH, 3OQI, 3OQ, 3NC6, 3NC7, 3NC3, 3NC5, 3N1A, 2X9Q, 3G5H, 3G5F, 1W1T, 1W1P, 1W1V, 1W1Y, 6VXV, 6VZB, 6WOS, 6VZA, 1O6I, *7OW9*, 6VZB, 6VXV, 6VZA. Interestingly, only one complex, 1QZR, contains 2,6-DKP core.

The Cambridge Structure Database

That notwithstanding, overwiew of the Cambridge Structure Database (CSD) to 531 (1070 without restrictions*) entries of 2,5-DKPs, 29 (43 without restrictions*) 2,3-DKPs and 37 (56 without restrictions*) hits of 2,6-DKPs. It is noteworthy that CSD collects a huge structural knowledge on potential peptide-based ligands that can be applicable at the macromolecular level. Small-molecular crystal structures, especially peptides and their derivatives, have a natural synergy with proteins. The rational design of modern effective ligands should be based not only on the 3D-structure of macromolecular target but also on potential ligands.

**restrictions: non-disordered, no errors, not polymeric, only simple crystal structures, only organics*

Table S1. Selected proline-based DKPs and their derivatives retrieved from the CSD (ver. May 2021).

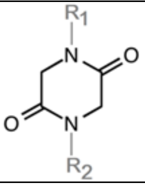	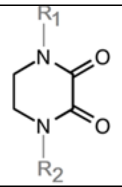	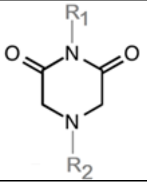

AKUWUY 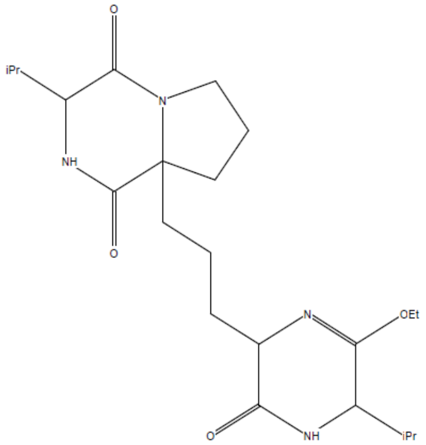	RUCDAW 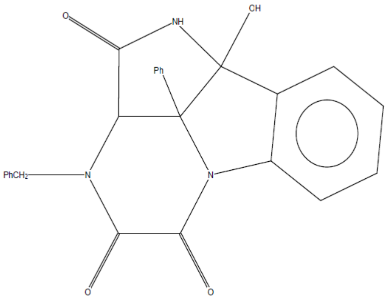	YIPRUK 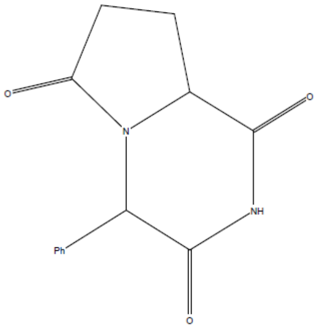
APUHOJ 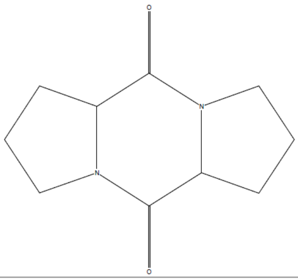		
BAMCEZ 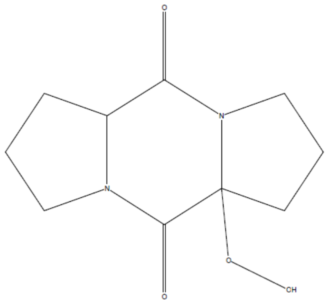		
**BIBKAA** 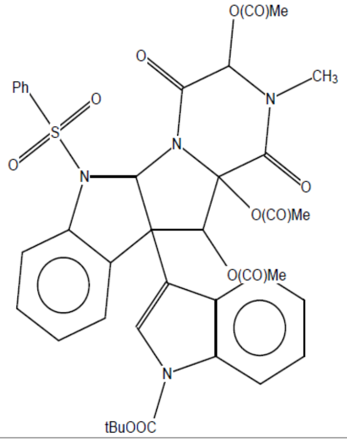		
**BIDDOJ**		

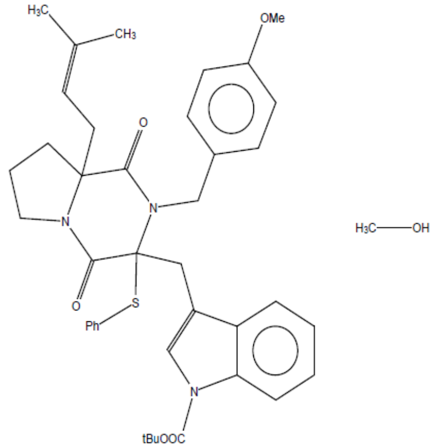		
**BONROL**<br>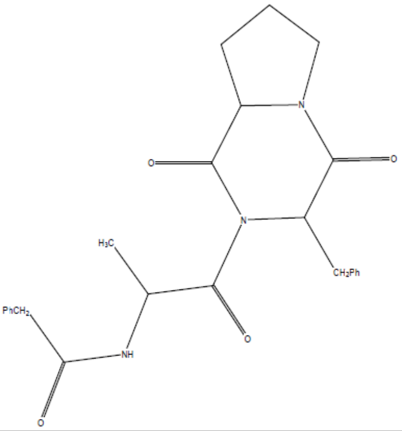		
**BOLWEE**<br>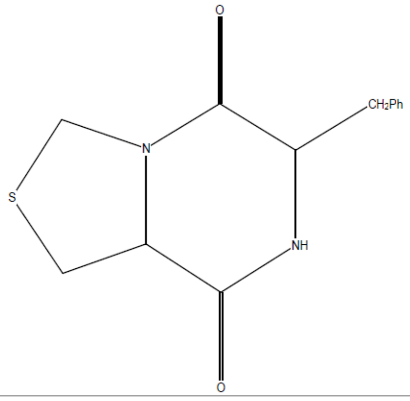		
**CAETOC**<br>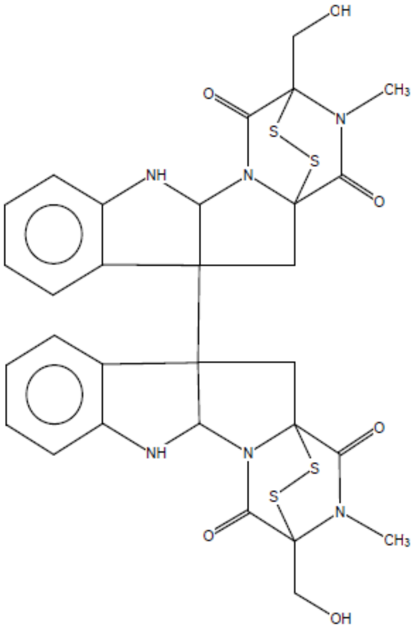		
**CEWBUE**		

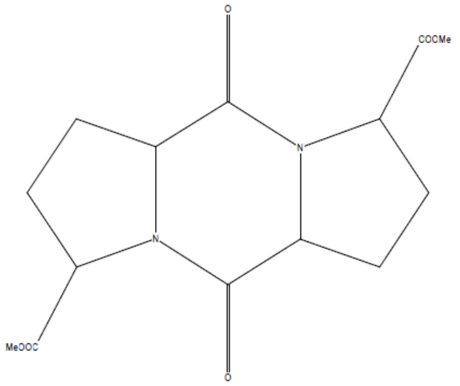		
CITXAE 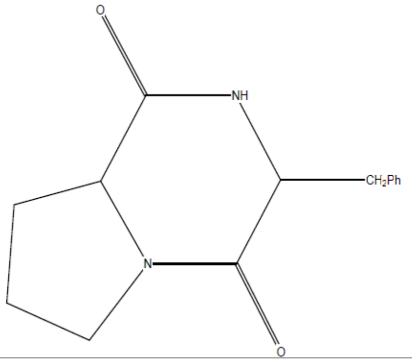		
CLPRAL01 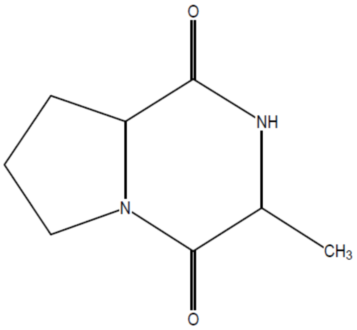		
CLPRPR01 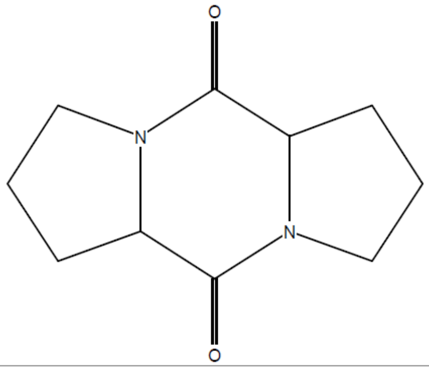		
COCYEZ		

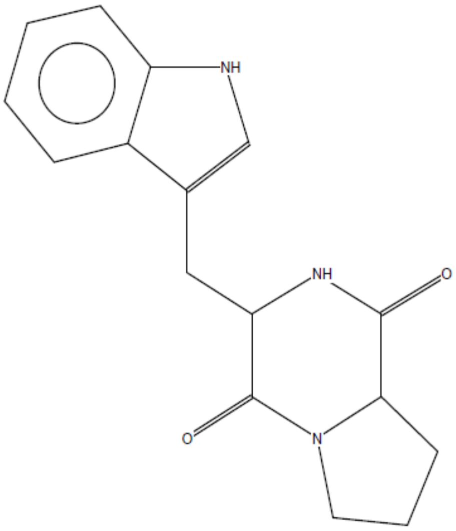		
COSGEX<br>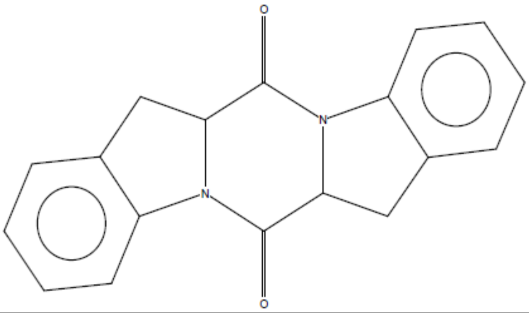		
**COYJIJ01**<br>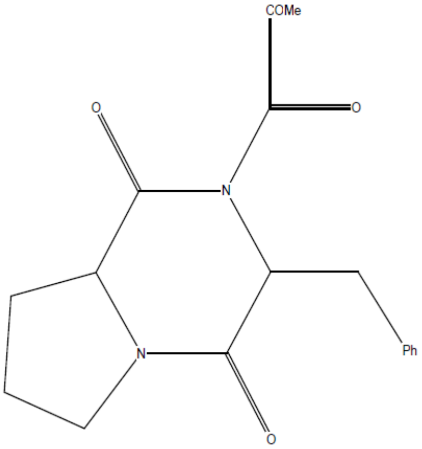		
CUWPER<br>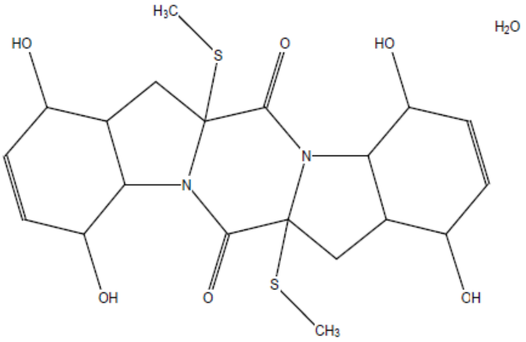		
**CYPAPR**11		

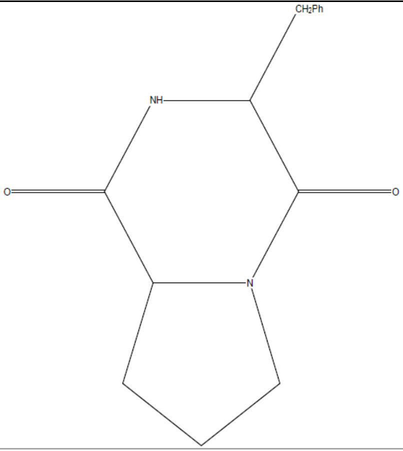		
**CYPRLE** 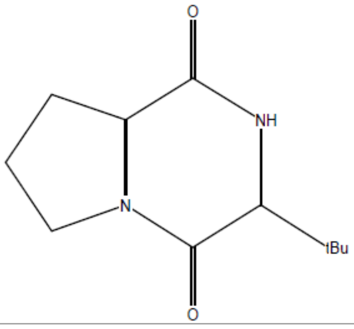		
DAGPAE 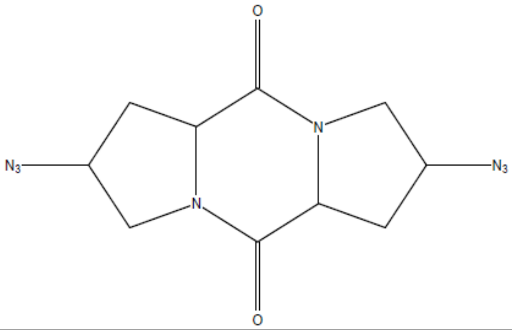		
**DARVID** 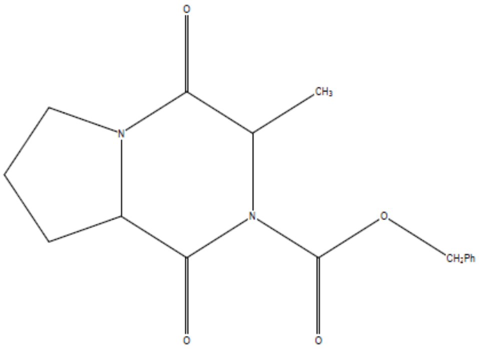		
**DARVOJ** 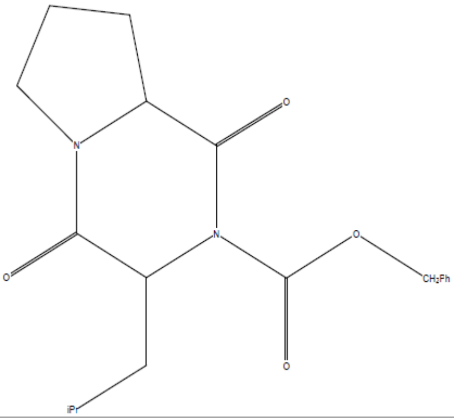		
DIGPEO		

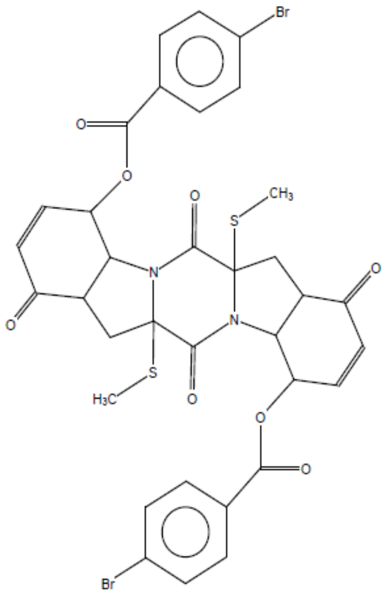

DITGIZ

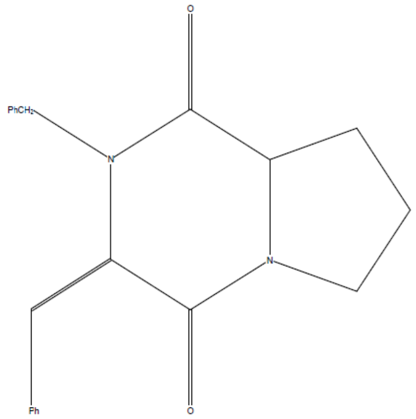

DUNVIS

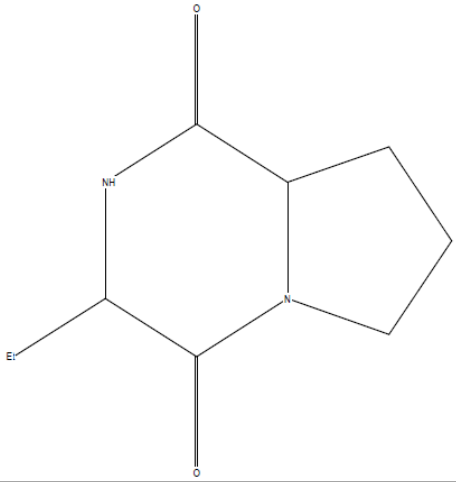

EBIFAZ

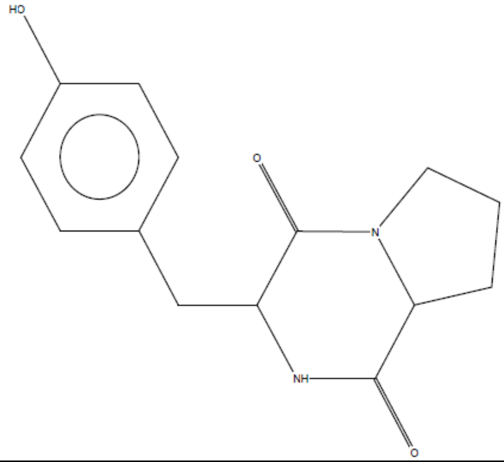

EJADUP<br>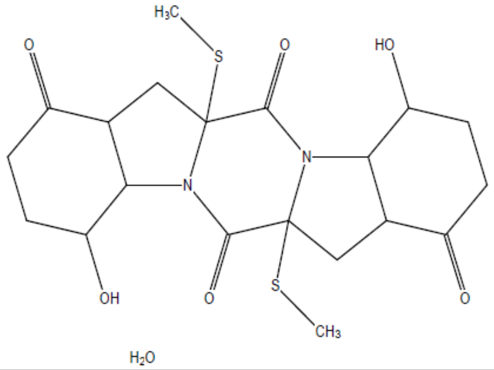		
EJADOJ<br>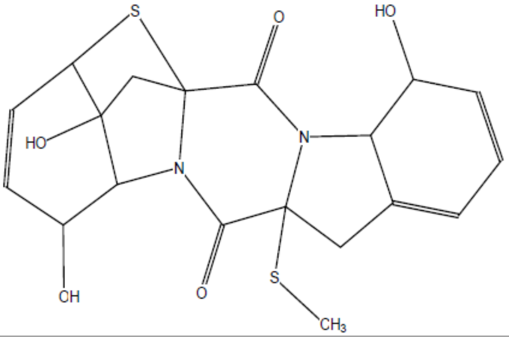		
EPICZA<br>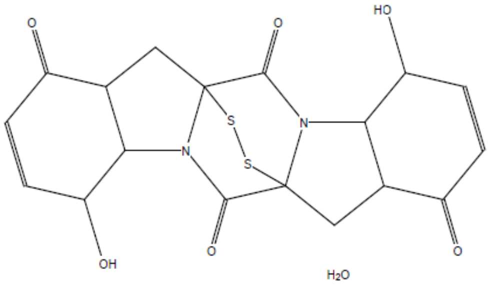		
**EXURAQ**<br>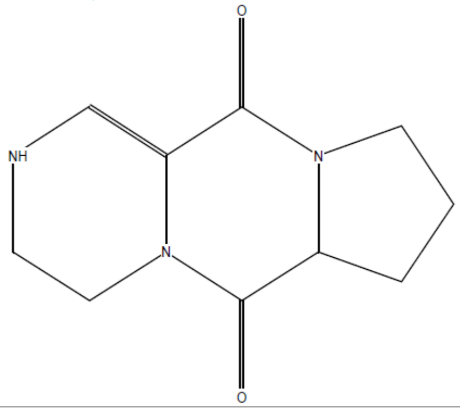		
**FEMWAV**<br>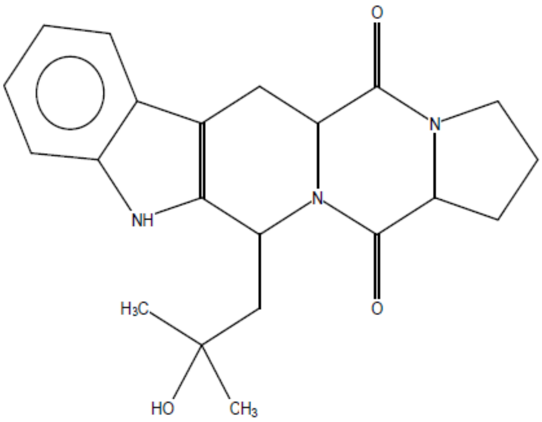		
**FEXXAH**		

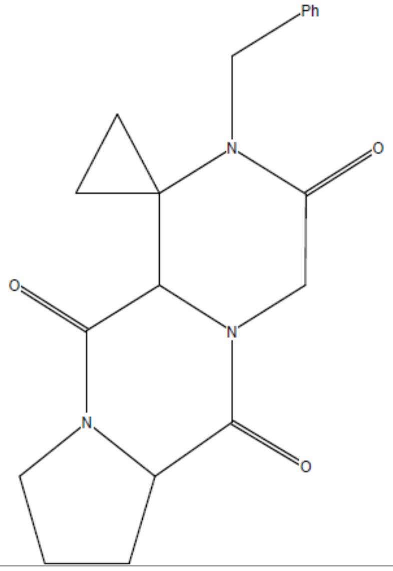		
FIFGAC01<br>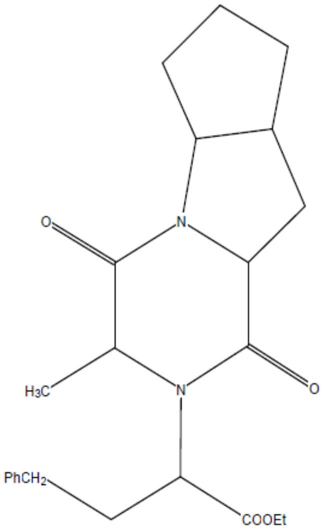		
**FODNES**<br>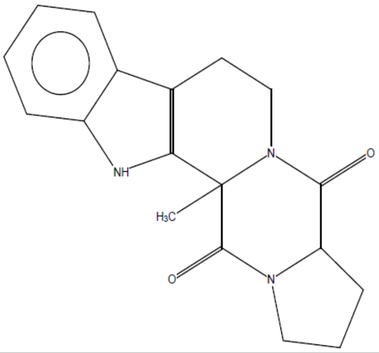		
**FODNOC**		

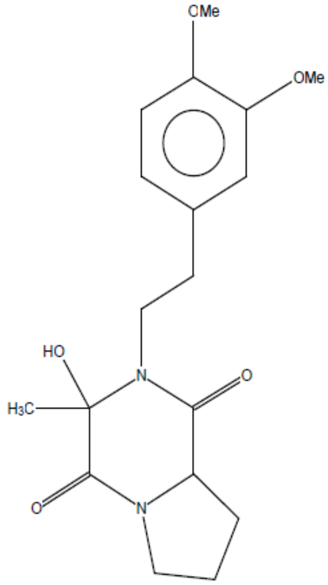		
**FODNUI** 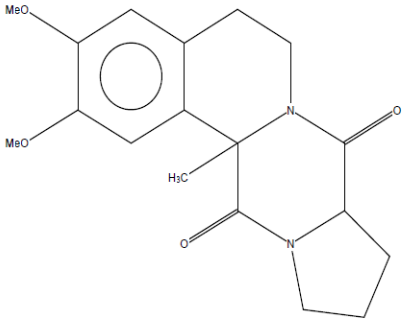		
**GESYAH** 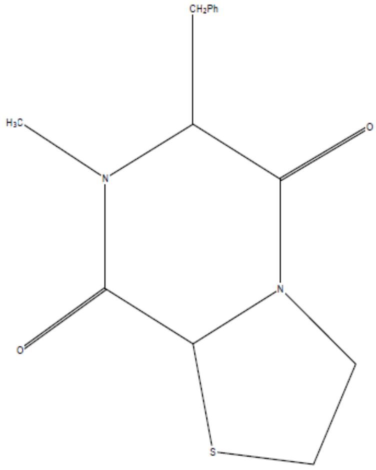		
**GESYEL** 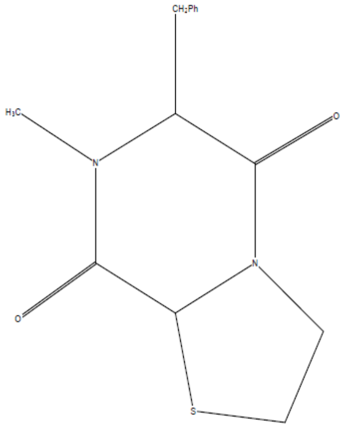		
**GESYIP**		

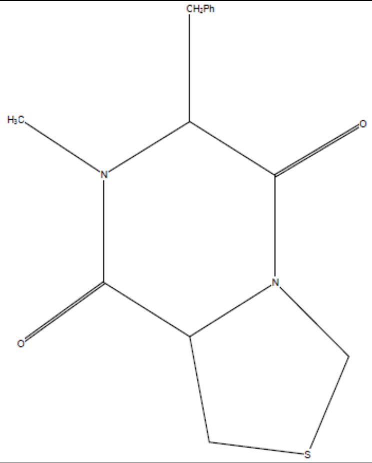		
**GESYOV** 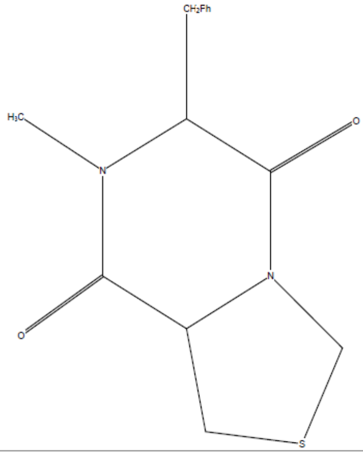		
**GLHPRD** 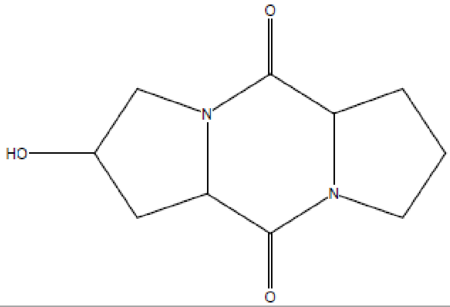		
**GUGXOW** 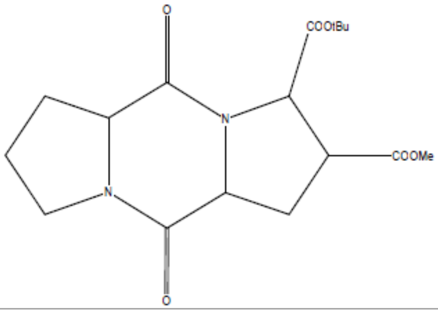		
HAMPAP 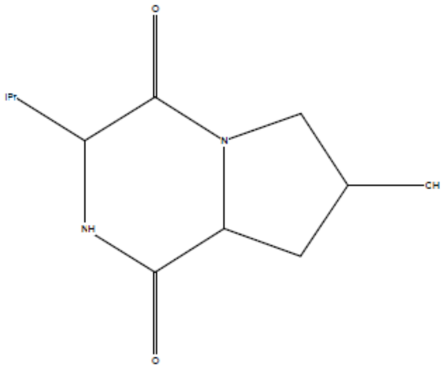		
HANJIR		

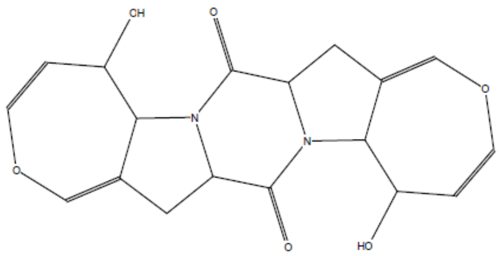		
HANJOX 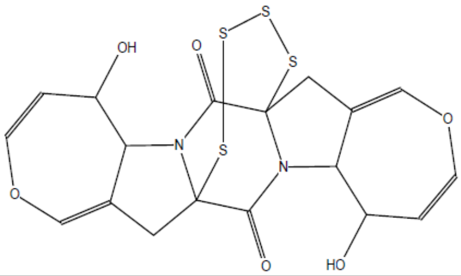		
HANJUD 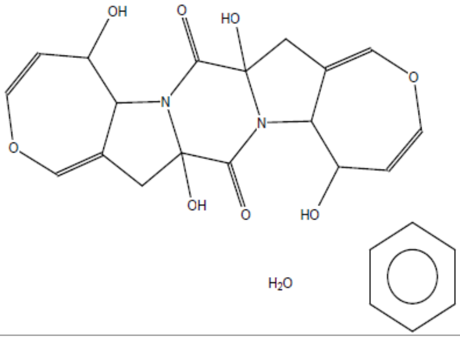		
HMTKIN 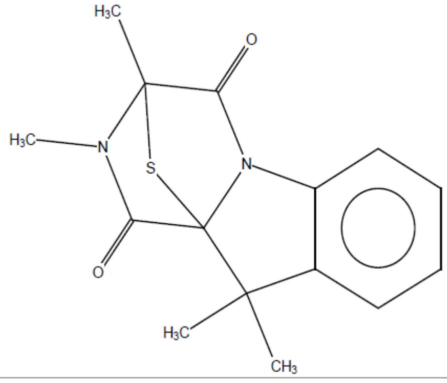		
HOYDAB 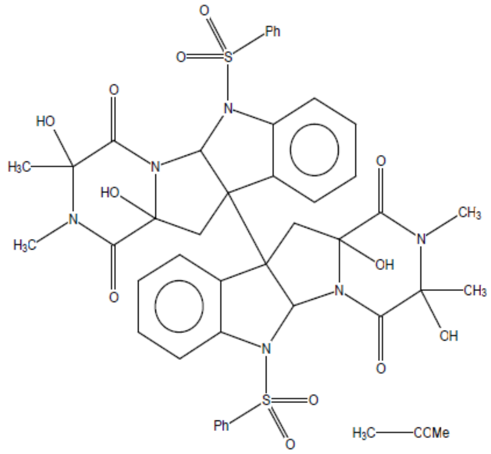		
HURCAA		

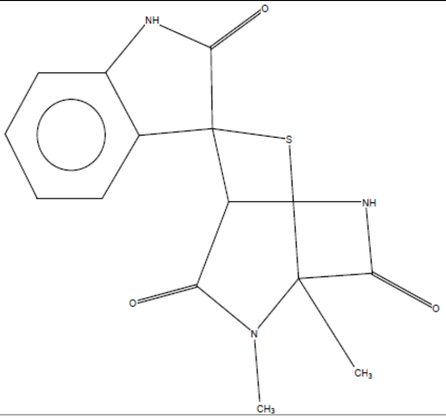		
IGAKOT 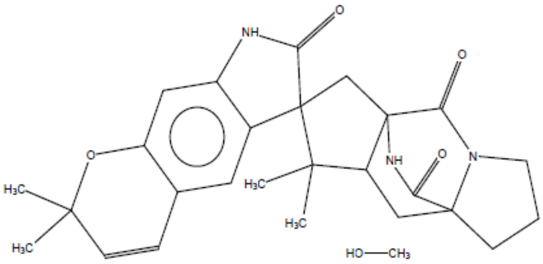		
IGODEP 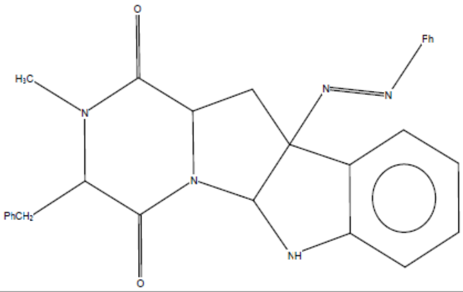		
IMIHAP 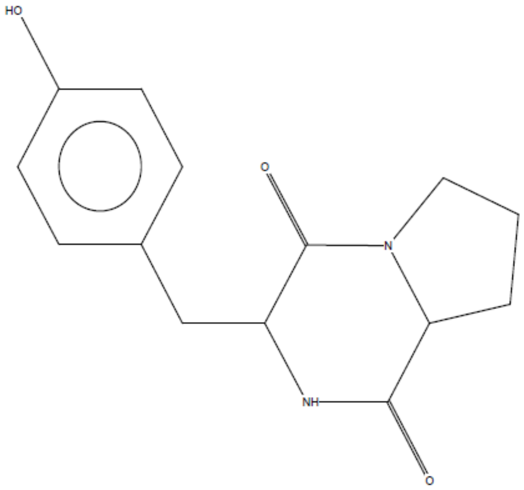		
IPIJUO 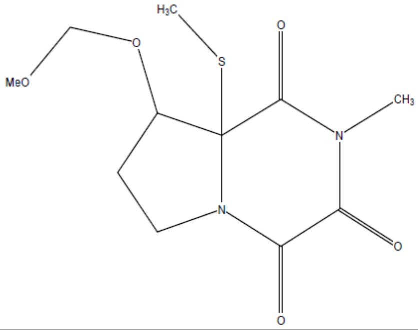		
IPIKAV		

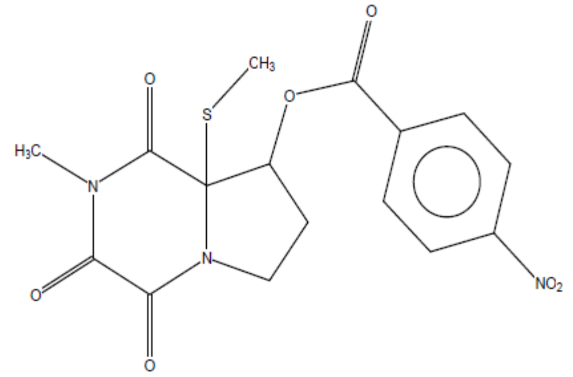		
IQUNIT 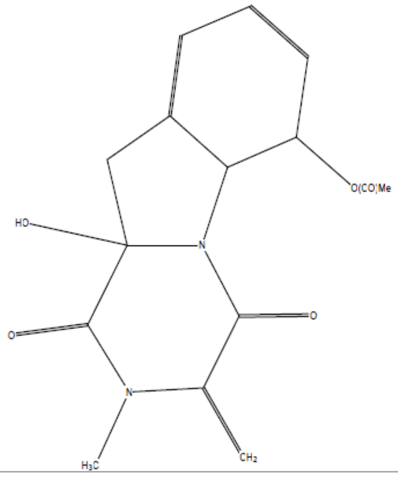		
ISUTIA 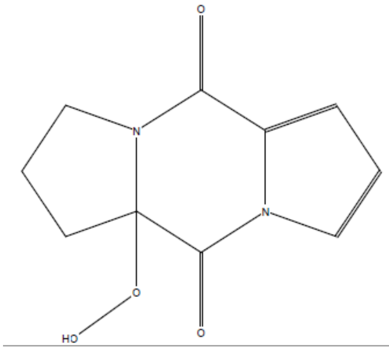		
ISUTOG 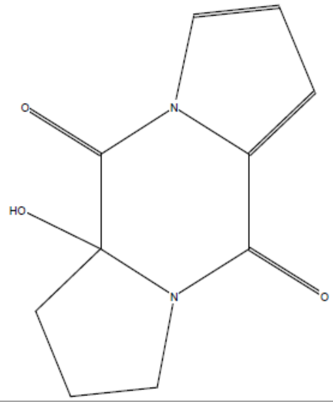		
**JAFFEC**		

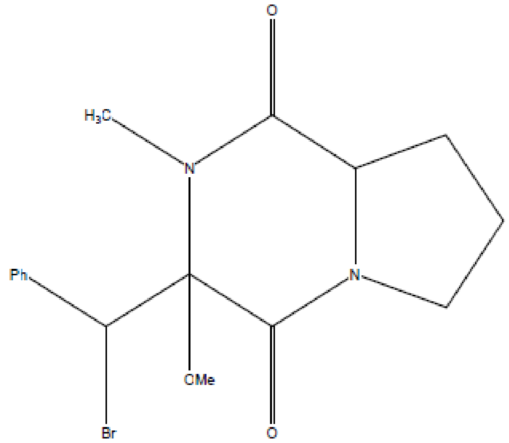		
**JEHPUH** 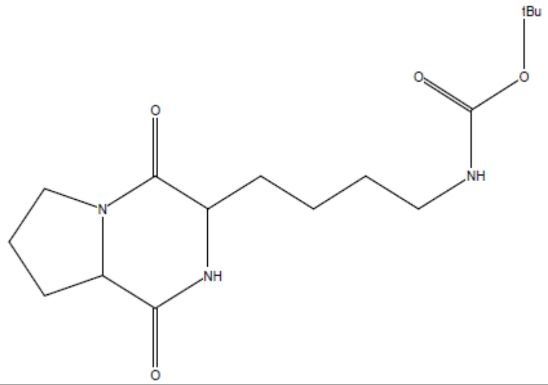		
**JIFNER** 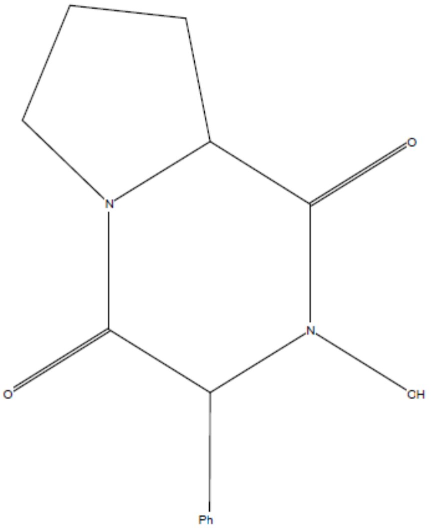		
**JUCSAB01** 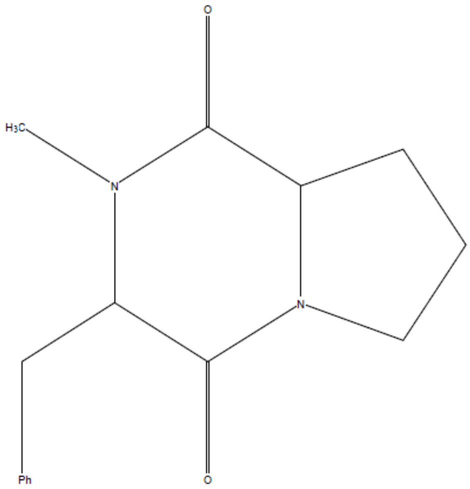		
KAQGIU01		

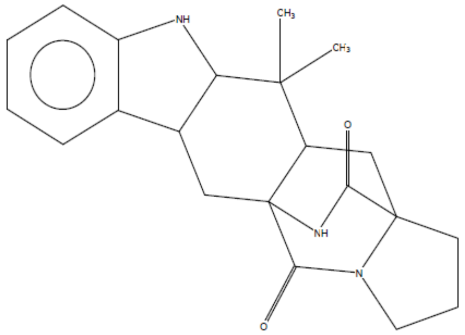		
KAQWOQ 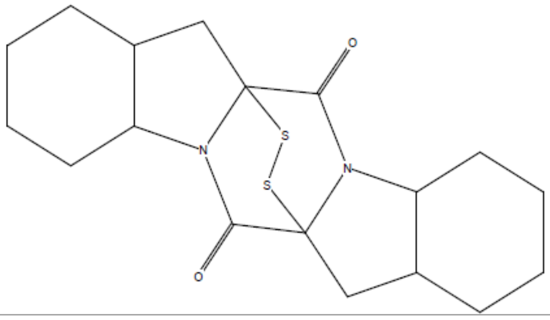		
**KAQXAD** 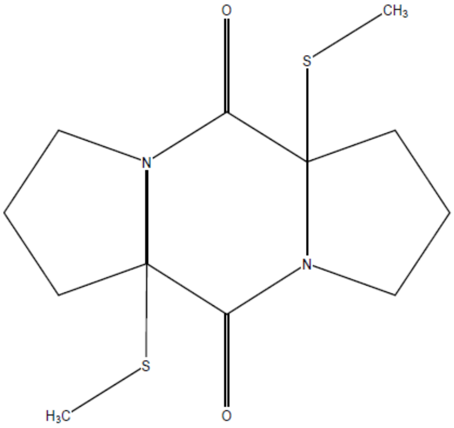		
KASJAR 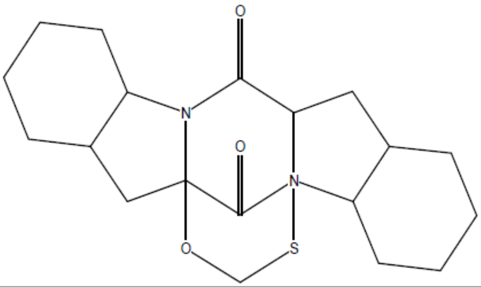		
**KETMIG01** 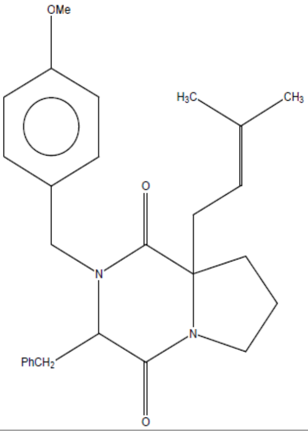		
KINLUO		

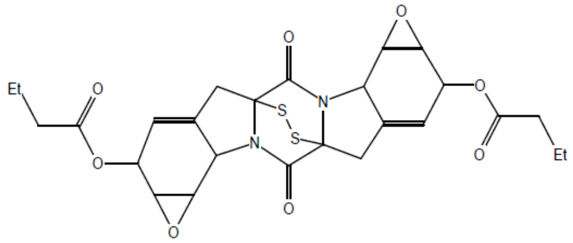		
**KUSXIF**<br>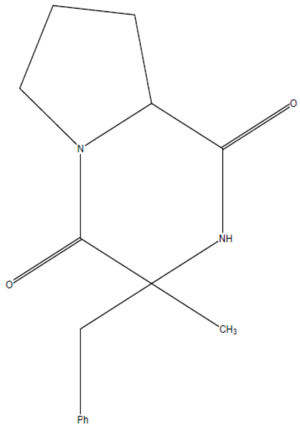		
**KUXJES**<br>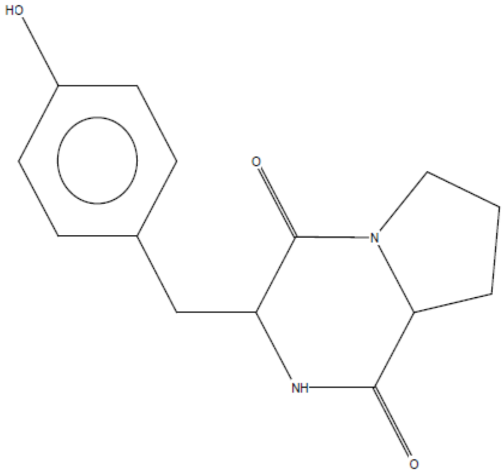		
**LEHREW**<br>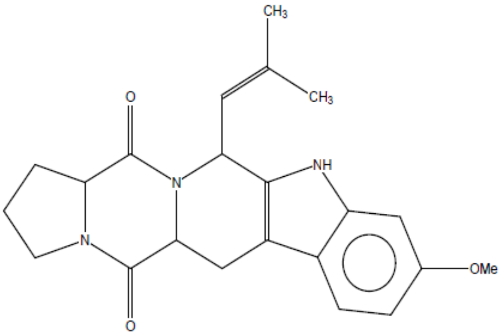		
LIHHUH		

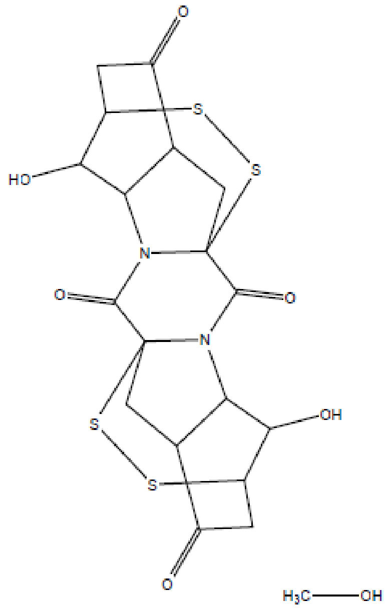		
LIHJAP 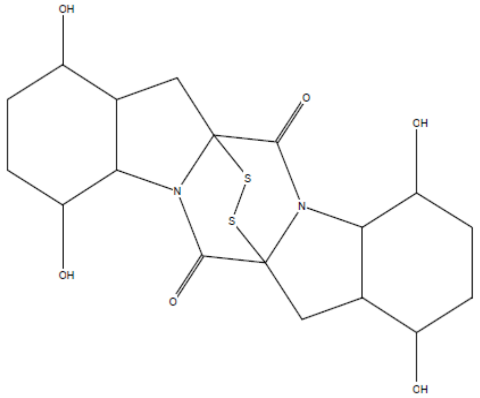		
**LIKNUO** 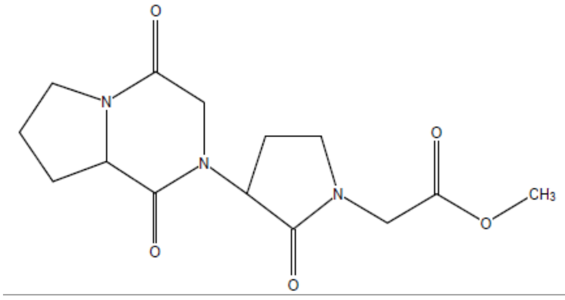		
LIMMUR 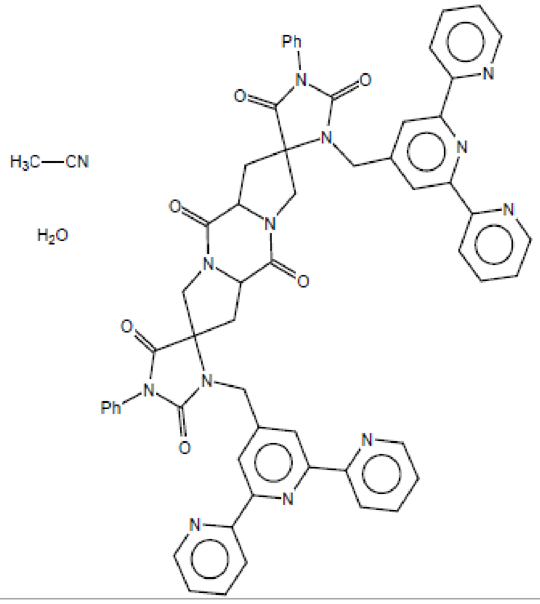		
**LPROGL**		

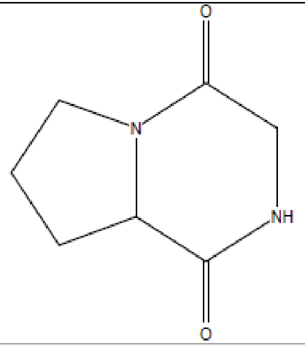		
MBTZCU<br>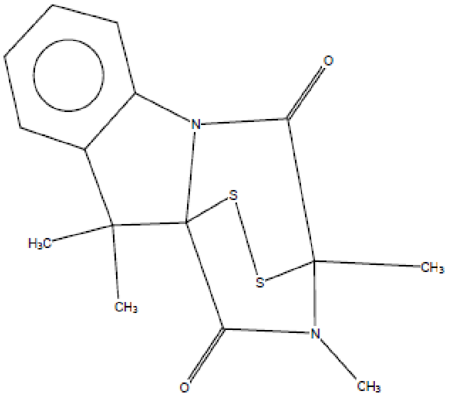		
MEMWUZ<br>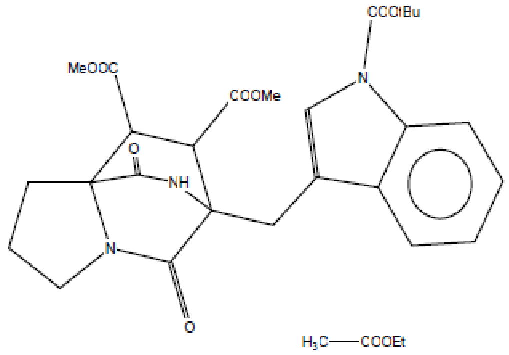		
MKMPIN<br>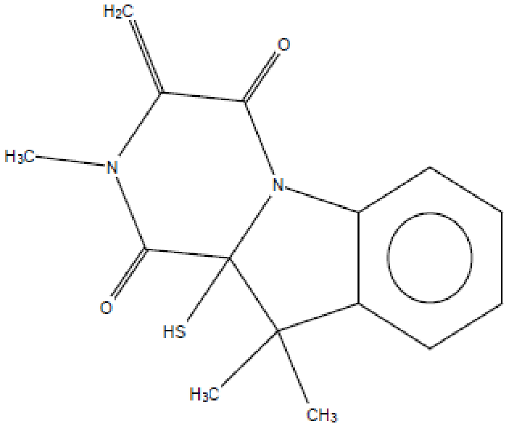		
MIRFAU		

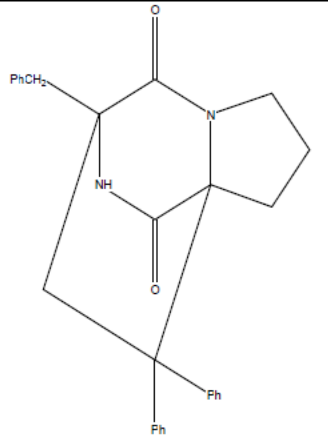		
MIRFEY<br>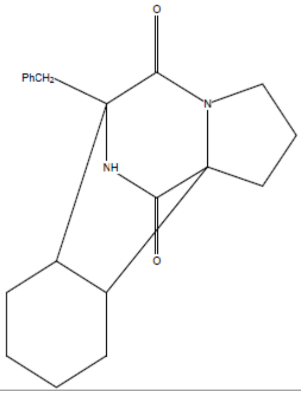		
MOPQUF<br>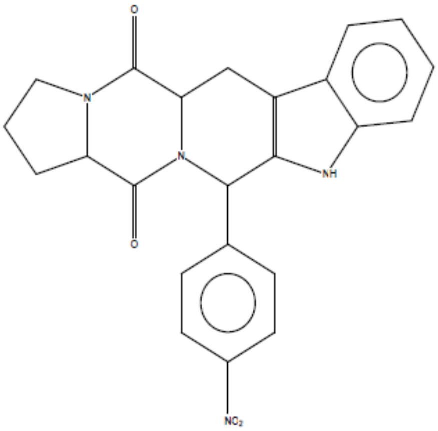		
MURSID<br>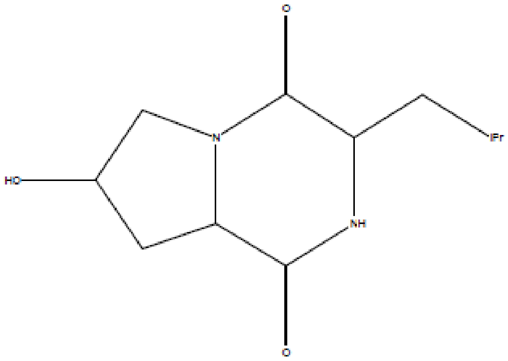		
MUSTUQ01<br>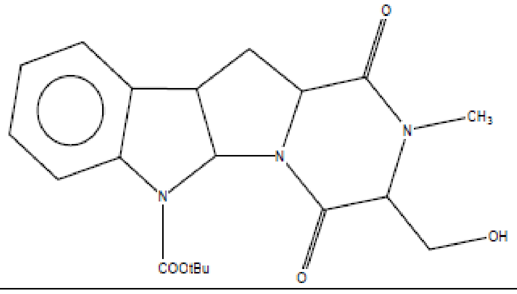		

NAHPUJ<br>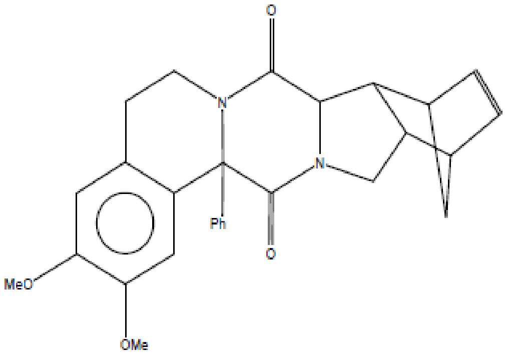		
NELFES<br>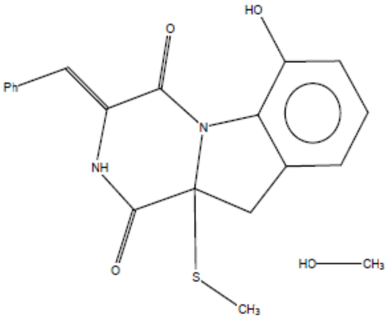		
**NEYLIP**<br>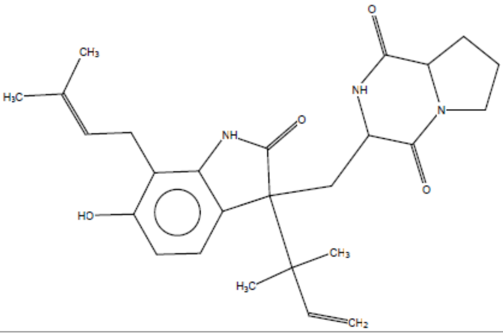		
NIVJIM<br>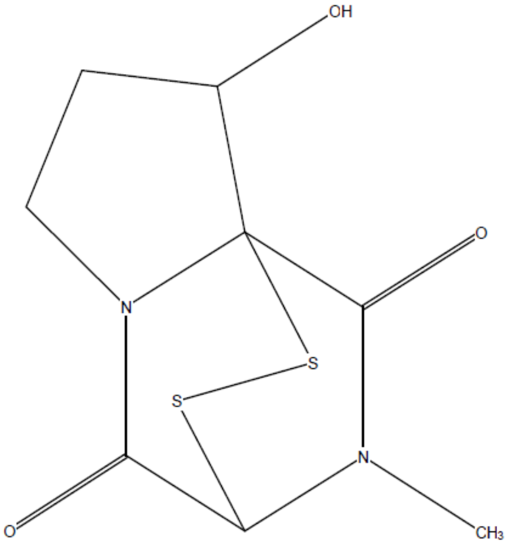		
**NOVCIK**		

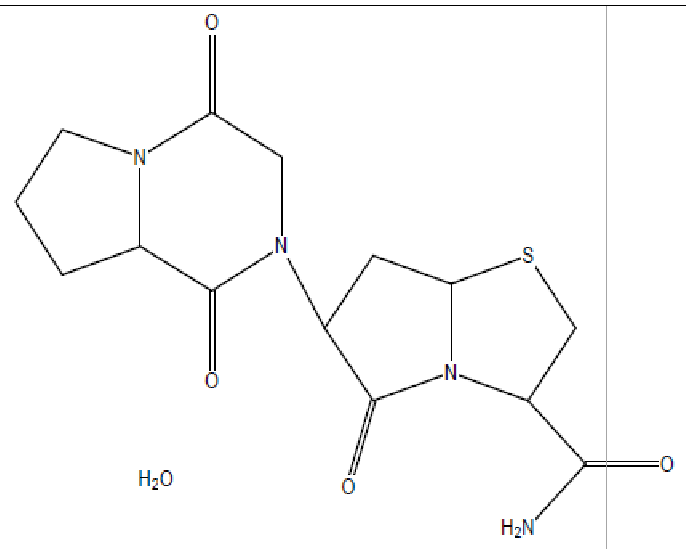		
NUKFAD 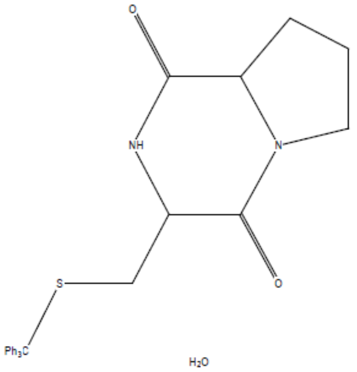		
ODALUD 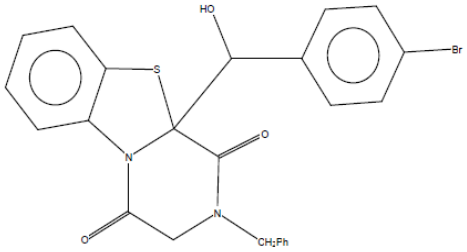		
ODIQUN 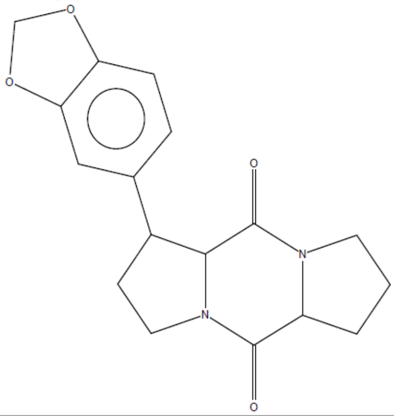		
OJECIR		

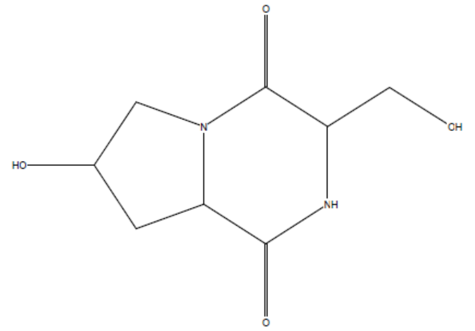		
OSULEU<br>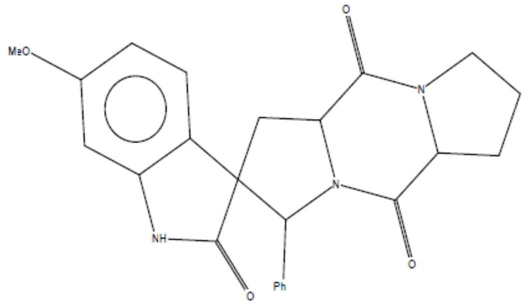		
PABRAM<br>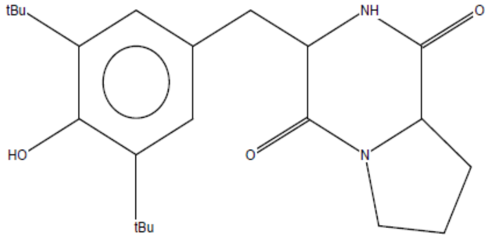		
PALLUJ<br>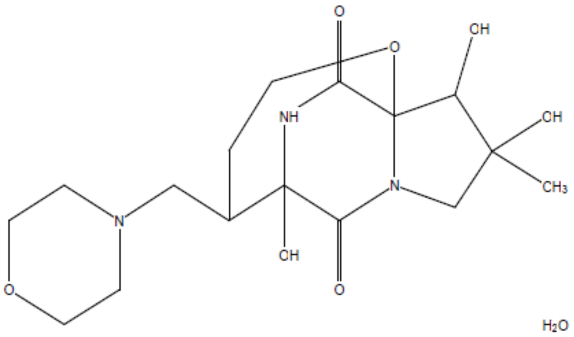		
PESYUK01<br>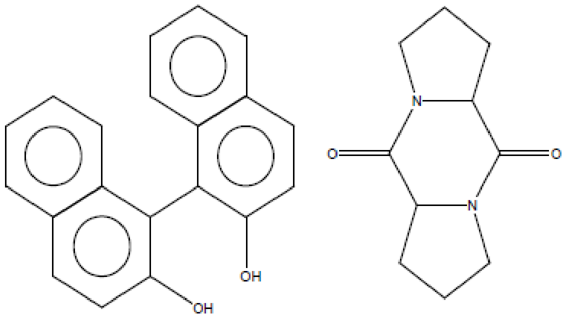		
PUTQOM<br>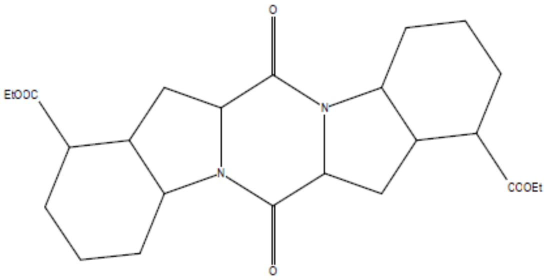		
PUYNUS		

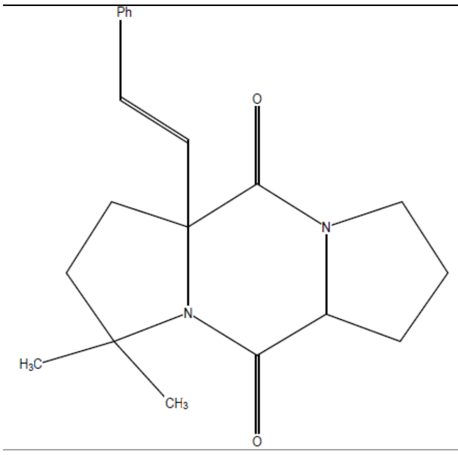		
QALPEA 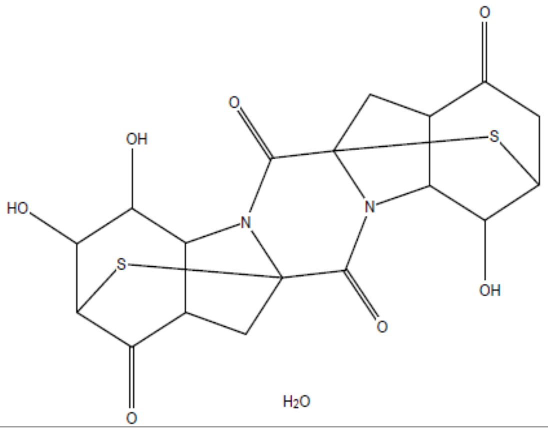		
QAXFIH 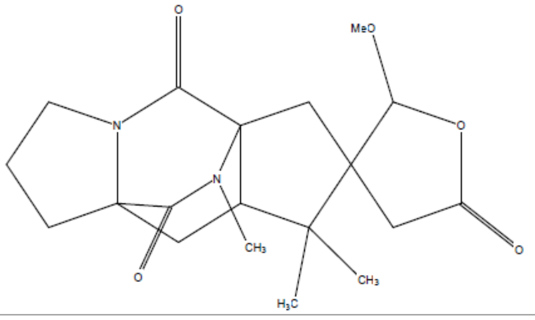		
QAXFON 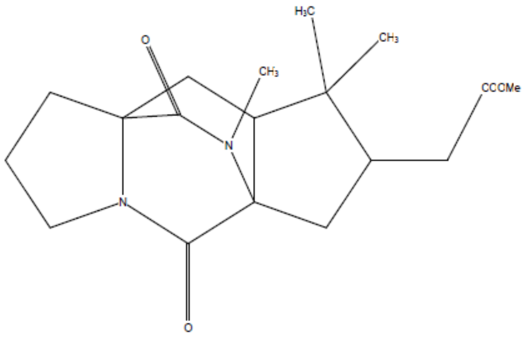		
**QAXFUT** 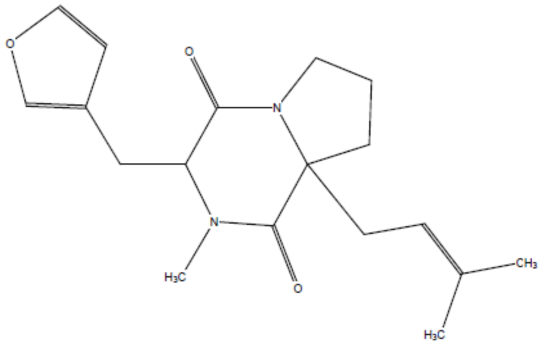		
QAXHEF		

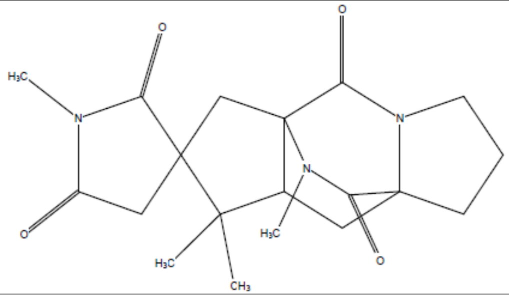		
**QELMID** 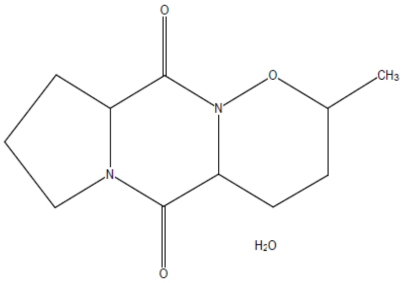		
QIBHAM 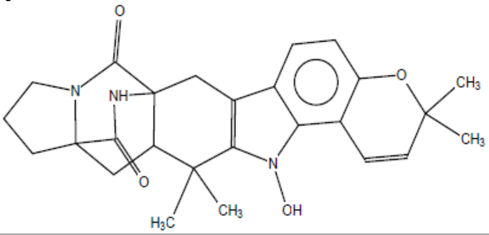		
**QOJXIY** 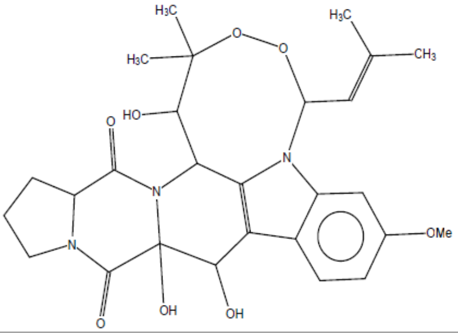		
**REFYIK** 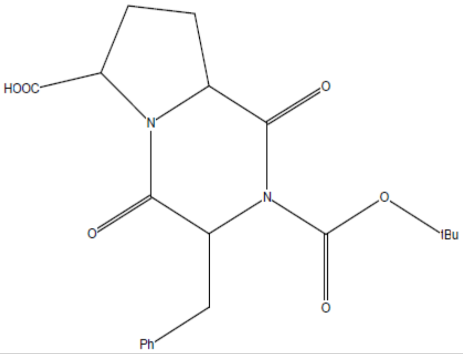		
RELNUU 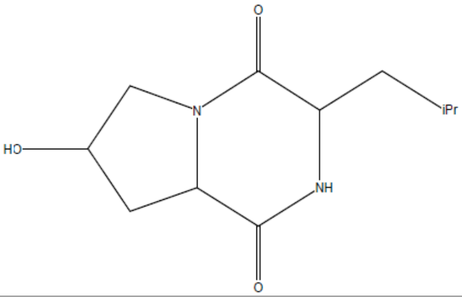		
REQLEF		

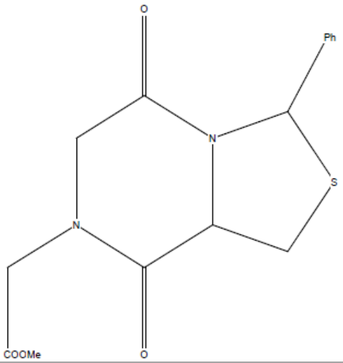		
REVVEW01 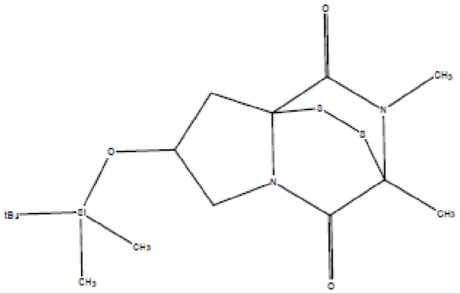		
**RIMTUE** 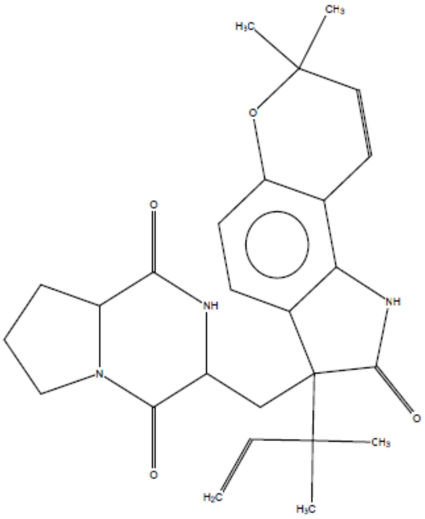		
**RISDIH** 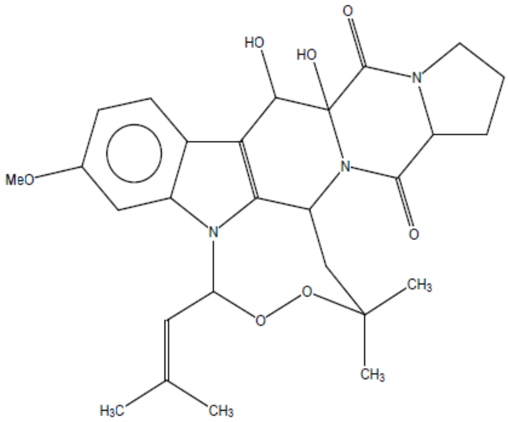		
**RIXXIG**		

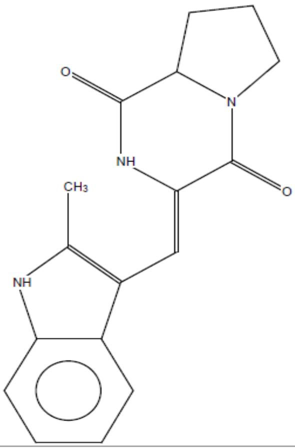		
RONJIQ 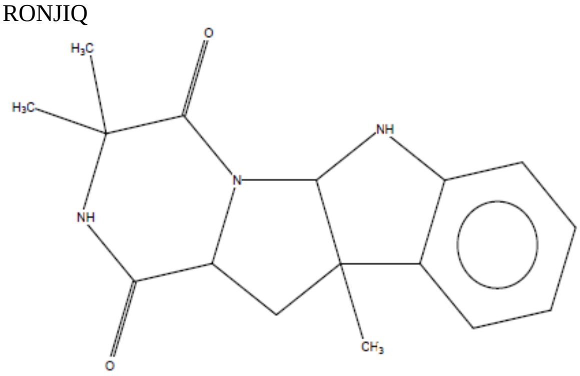		
RONJOW 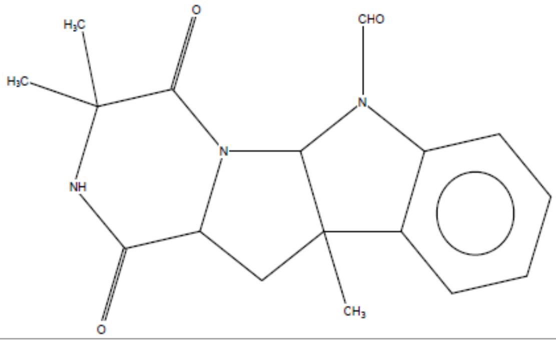		
**ROPVOH** 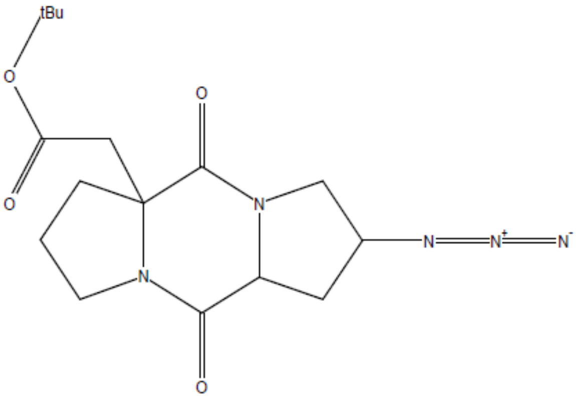		
SEDROL 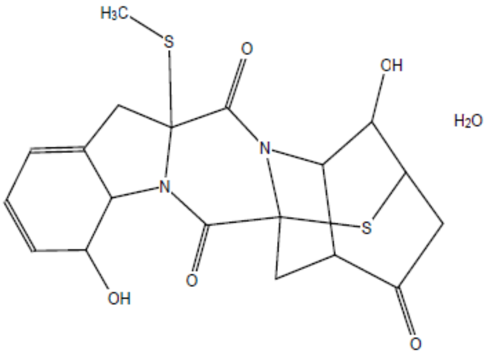		

SEDSAY 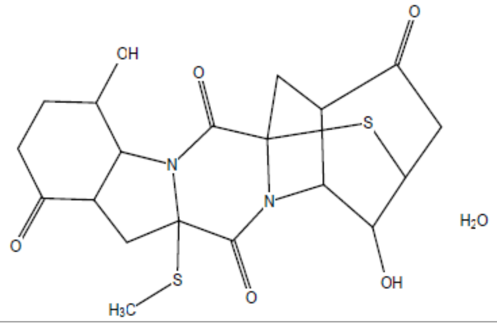		
SEDSIG 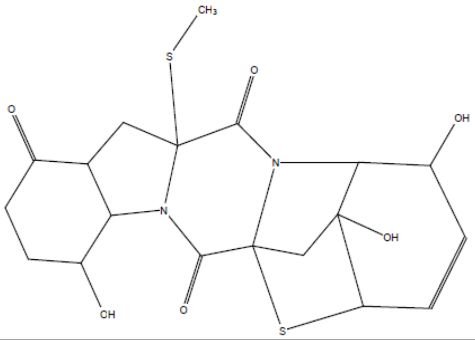		
SEPFAU 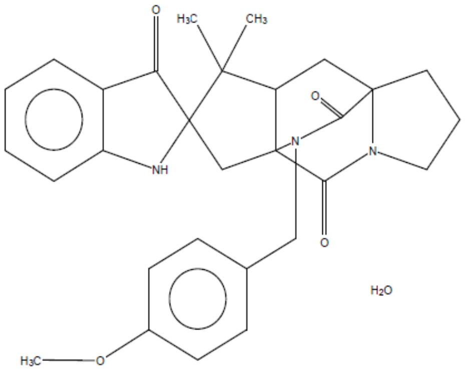		
SEPFIC 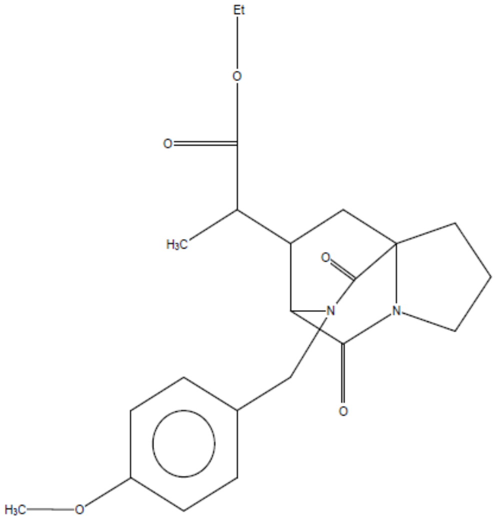		
SEPFOI		

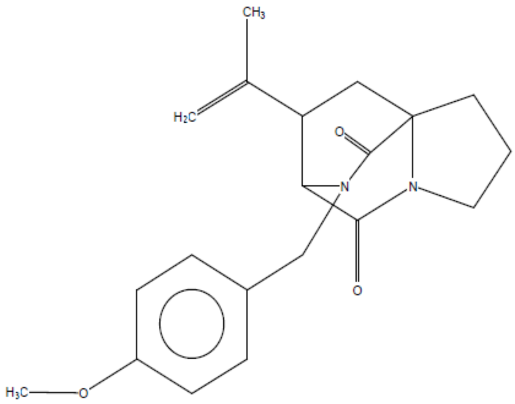		
SIRPLA 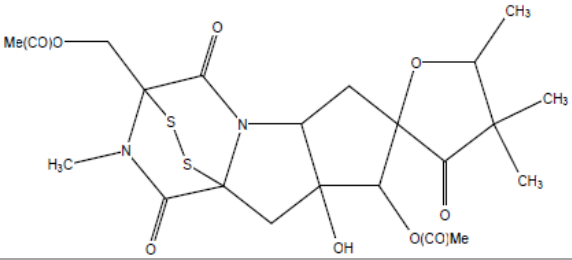		
SUGWUM 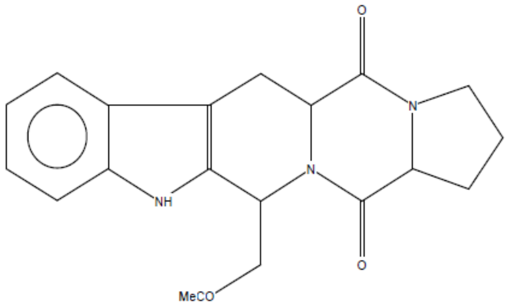		
TAYJOU 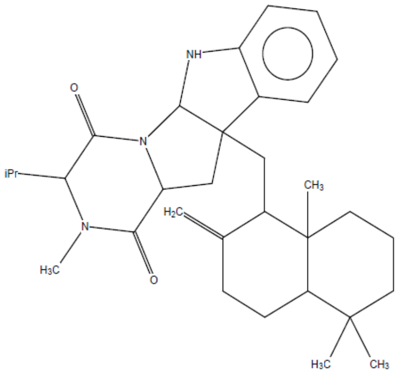		
TIBCIR 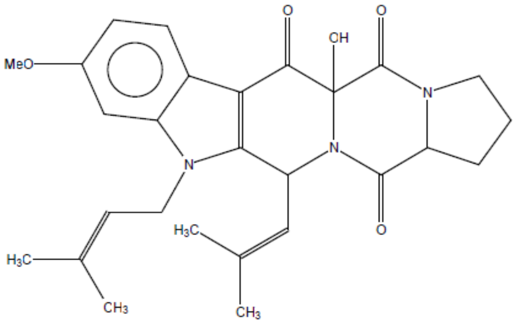		
TIMHUT		

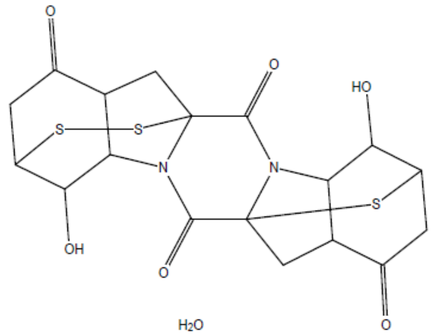		
TOJQEQ 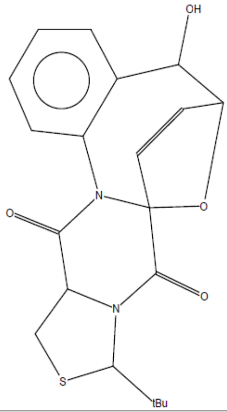		
TOJQIU 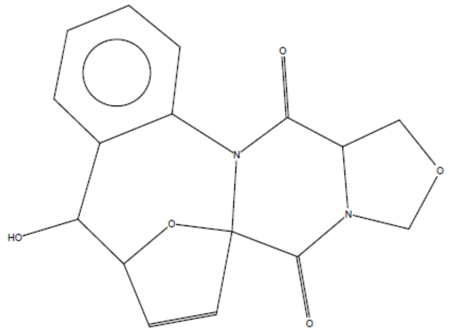		
TOJQOA 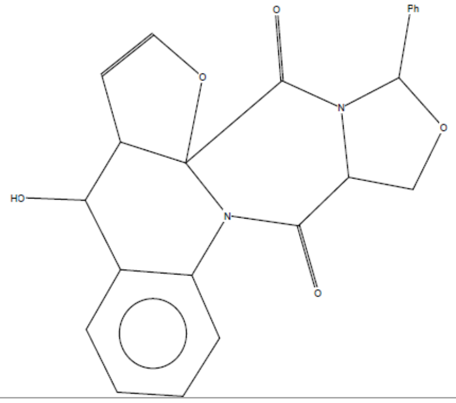		
TOJQUG 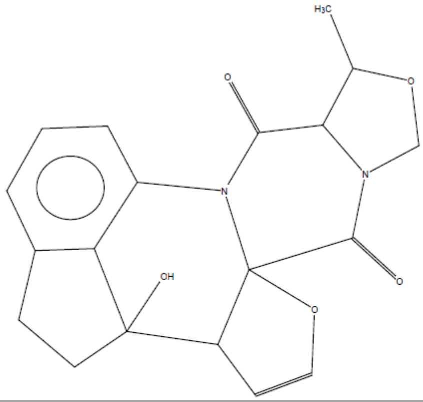		
TOJRAN		

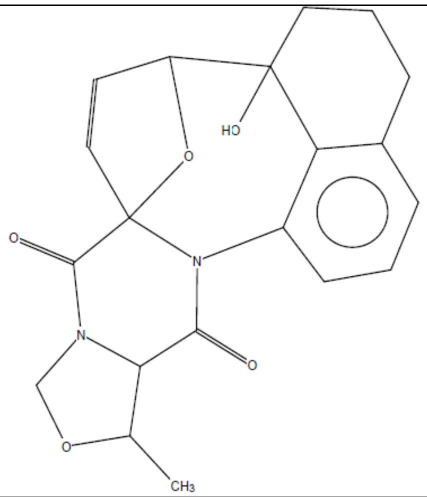		
TOJRIV 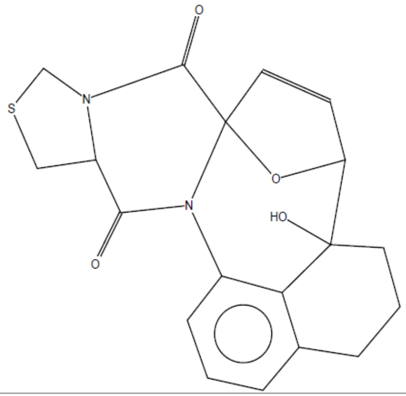		
TUNZAF 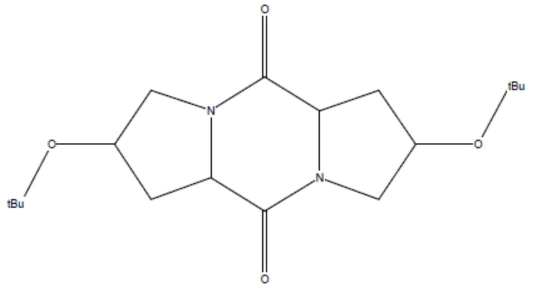		
TUNZIN 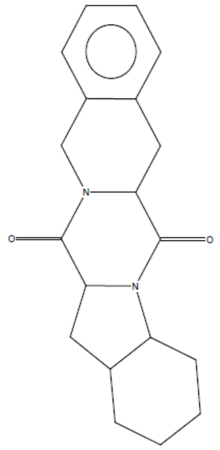		
TUNZOT 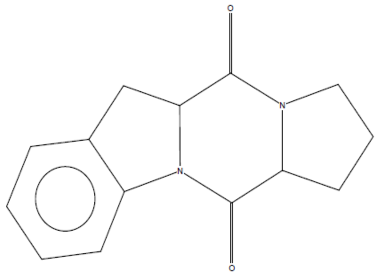		

TUNZUZ 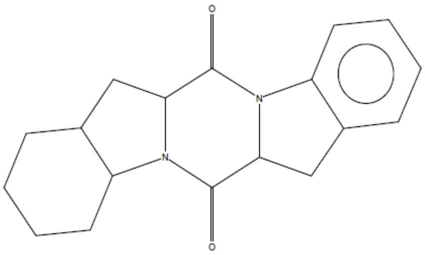		
TUPBEN 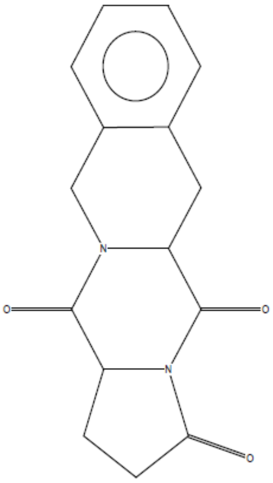		
TUPBUD 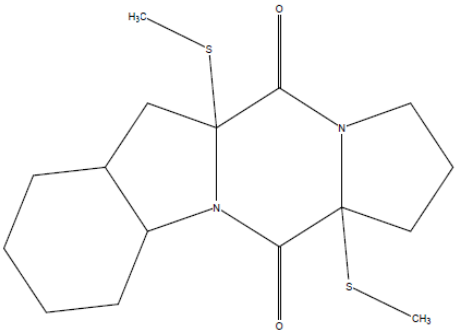		
TUPJIZ 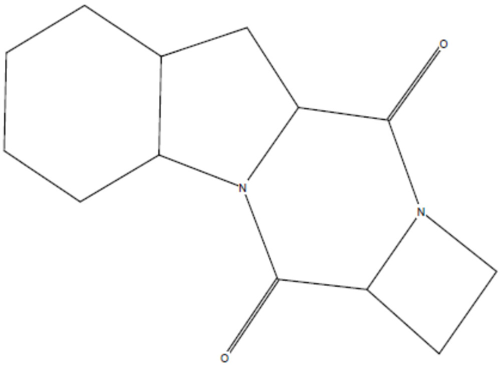		
**TUTSOR** 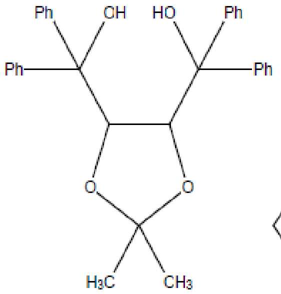 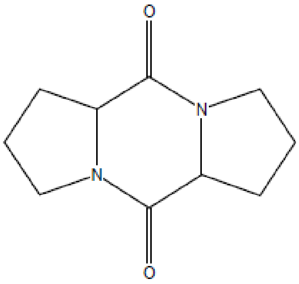		
**UCOBEV**		

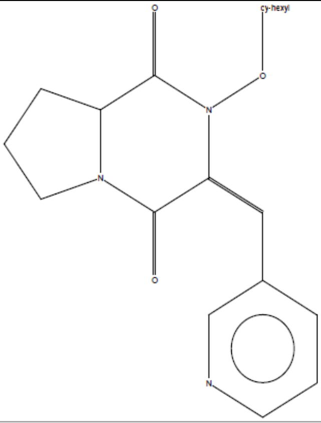		
**UDALUJ**<br>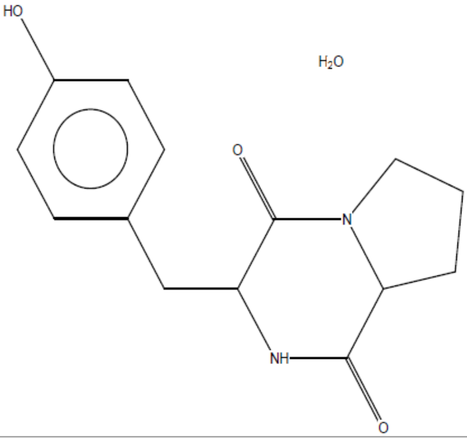		
UGOQAJ<br>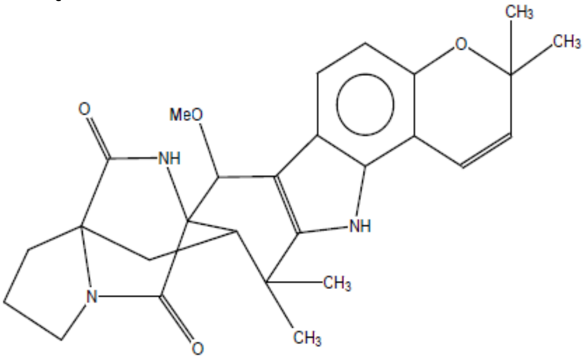		
**UKOCON**<br>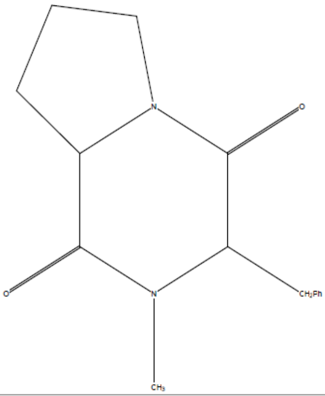		
UQIMEN		

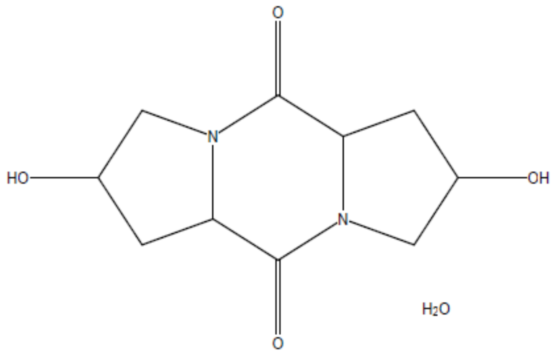		
VACPUL<br>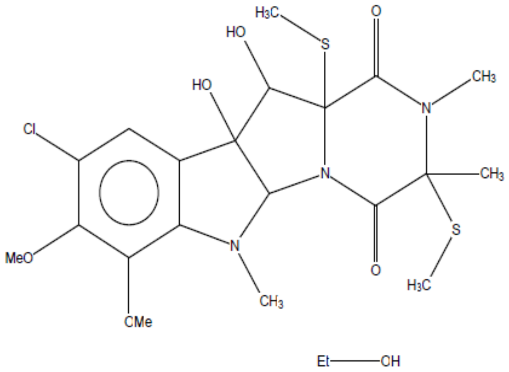		
VAVZUP<br>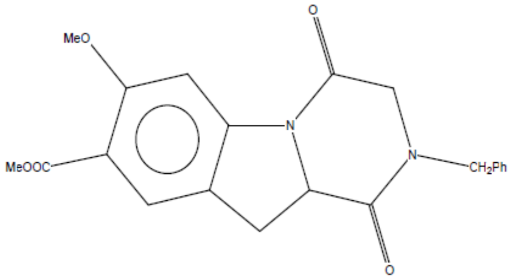		
VAZRAQ<br>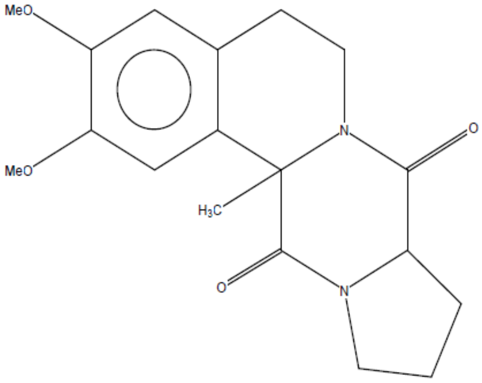		
VEVXEB<br>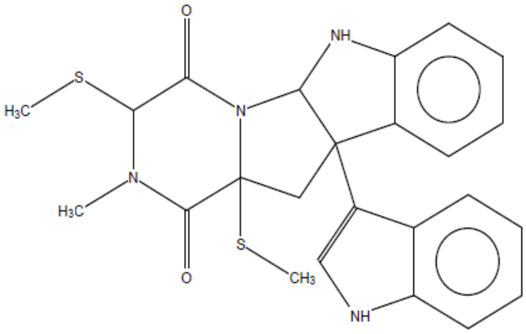		
**VIDRAB**		

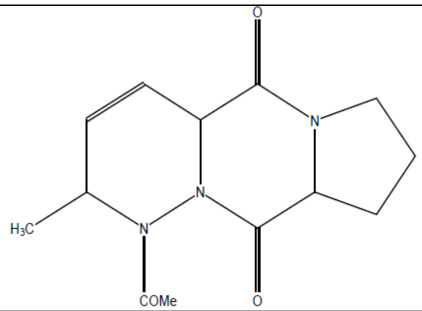		
WANTEN<br>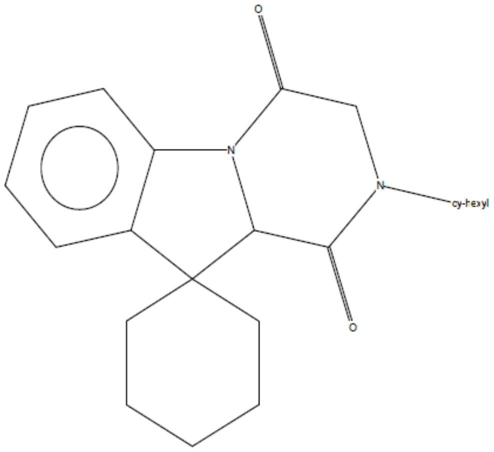		
WEWWAY<br>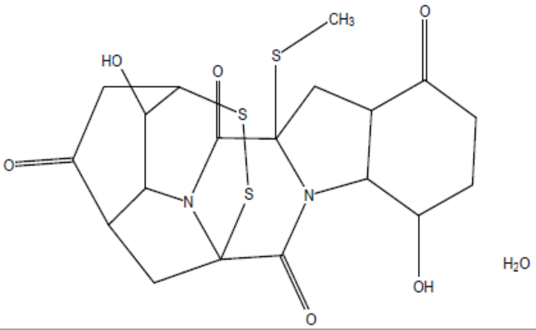		
XENVES<br>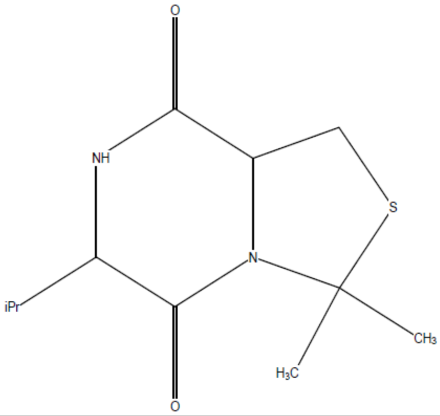		
XENVIW<br>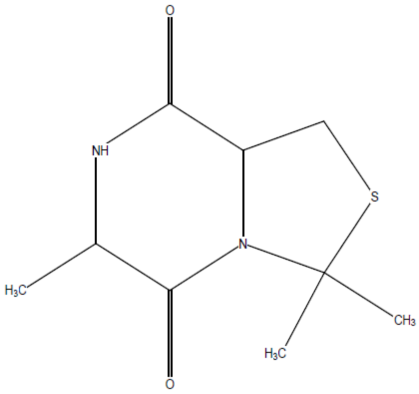		

XOLQUL 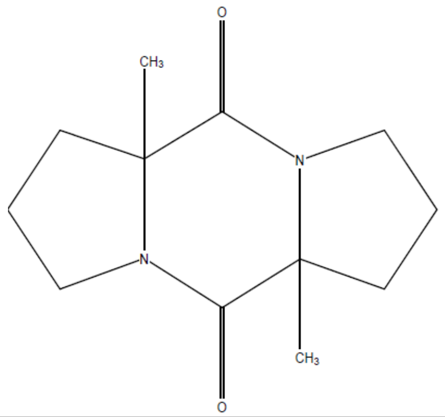		
XOYYOC 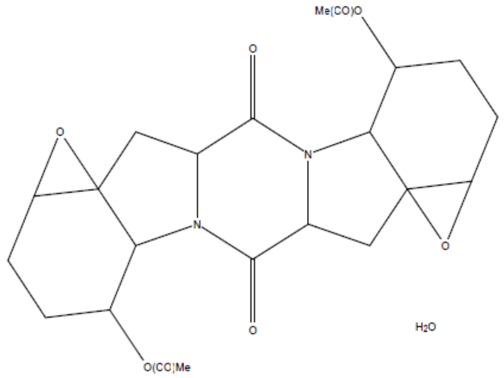		
XOYYUI 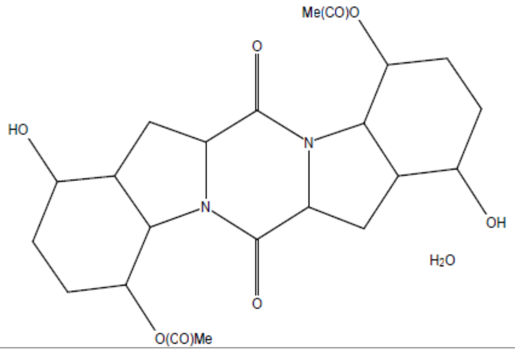		
XOYZAP 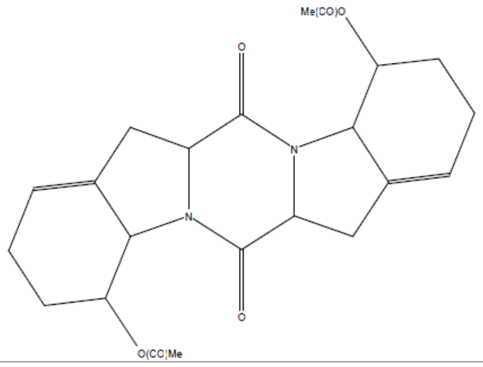		
XUMCIR		

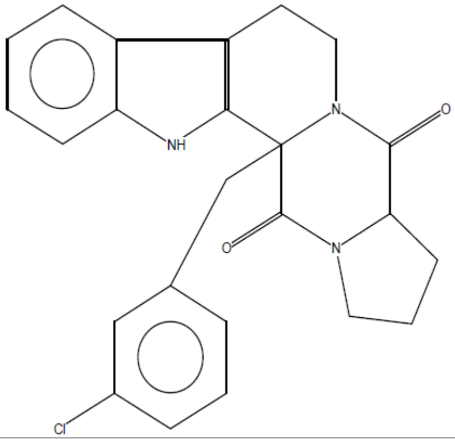		
YERPIU<br>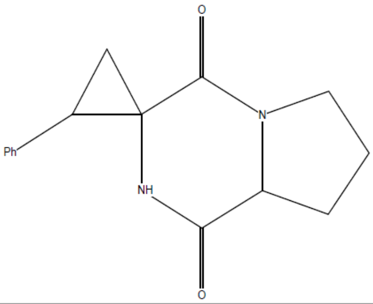		
YEYXAD<br>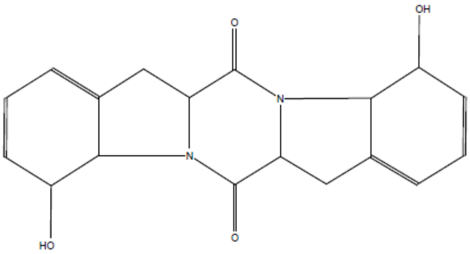		
YEYXEH<br>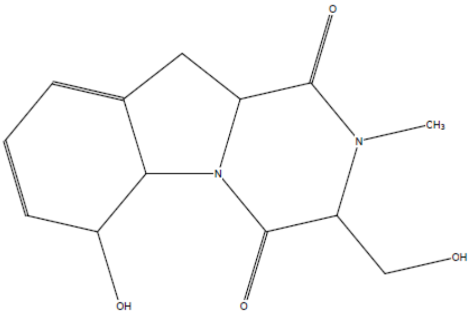		
YOMXII<br>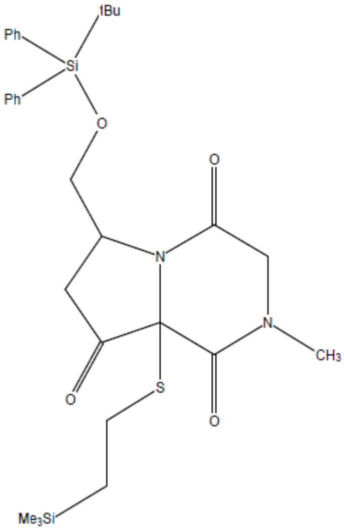		
**ZAJZOZ**		

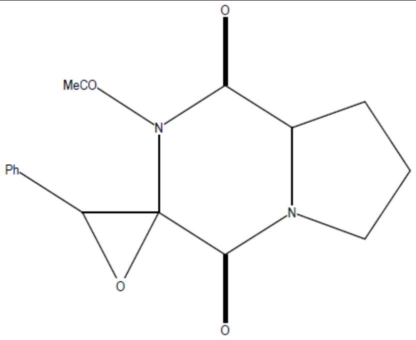		

Table S2. Graphical schemes of proline-DKP-based supramolecular synthons (CSD, ver. May 2021).

H-bonding Interaction forming synthon	*Synthons and CSD entries, containing these motifs*	*H-bonding Interaction forming synthon*	*Synthons and CSD entries, containing these motifs*
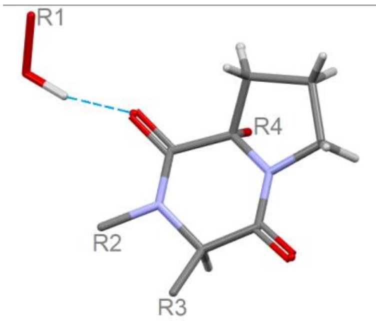	**D(2):**<br>BUDDOJ, FODNEC, PESYUK, TUTSOR, UDALUJ<br>**C(5)**:<br>QOJXIY, RISDIH<br>**C(6)**:<br>BAMCEZ, JIFNER<br>**C(8)**:<br>FEMWAV<br>**C(10)**:<br>IMIHAP, KUXJES	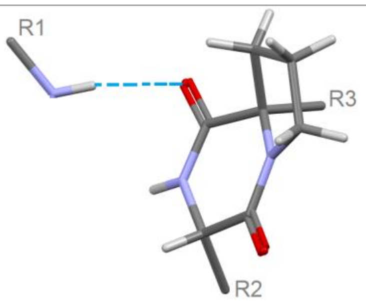	**C(4)**: PABRAM<br>**C(5)**: CYPRLE, DUNVIS, EBIFAZ, JEHPUH, LPROGL, UDALUJ, YERPIU<br>**C(6)**: CYPRLE, EXURAQ, OSELEU<br>**C(7)**: CYPRLE, MOPQUF<br>**C(8)**: LEHREW, NEYLIP, OSELEU<br>**C(9)**: OSELEU, RIXXIG<br>**C(10)**:<br>AKUWUJ, JEHPUH<br>**D(2)**:<br>EXURAQ, FODNES, KUSXIF, RIMTUE, RIXXIG<br>**D$^{2}_{3}$(9)**:<br>EXURAQ
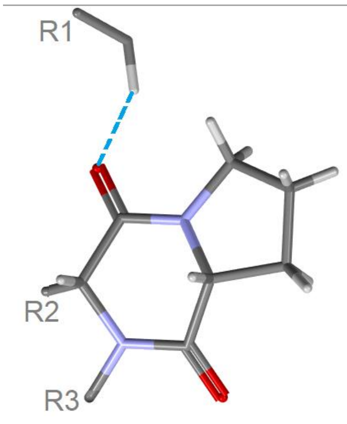	**C(4)**: CLPRAL, DARVOJ, FEXXAH, ODIQUN, QAXFUT, ROPVOH<br>**C(5)**: APUHOJ, BAMCEZ, CLPRPR, EBIFAZ, GESYAH, GESYEL, GESYIP, GLHPRD, JEHPUH, JUCSAB, KETMIG, LPROGL, PABRAM, PUYNUS, RIMTUE, ROPVOH, UKOCON, XOLQUL<br>**C(6)**: APUHOJ, BAMCEZ, CLPRAL, CLPRPR, FODNUI, GESYAH, GESYEL, GESYOV, GUGXOW, JIFNER, JUCSAB, KAXQAD, KUSXIF, LEHREW, LPROGL,	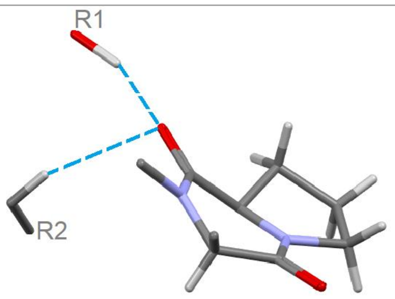	**C$^{1}_{2}$(8)**: JIFNER<br>**C$^{1}_{2}$(9)**:<br>BAMCEZ<br>**C$^{1}_{2}$(10)**: EBIFAZ

	MOPQUF, NEYLIP, ODIQUN, PABRAM, PUYNUS, QAXFUT, REFYIK, ROPVOH, UCOBEV, UKOCON, VAZRAQ, XOLQUL, YERPIU, ZAJZOZ<br>**C(7)**: FEXXAH, FODNOC, FODNUI, JAFFEC, JIFNER, MOPQUF, NEYLIP, NOVCIK, LIKNUO, PUYNUS<br>**C(8)**: BUDDOJ, DARVID, EBIFAZ, FODNUI, JIFNER, KETMIG, KUXJES, ODOQUN, PUYNUS, QAXFUT, YERPIU<br>**C(9)**: DITGIZ, FODNES, GESYEL, GESYIP, GUGXOW, KETMIG, KUSXIF, KUXJES, MOPQUF, REFYIK, RIXXIG, ROPVOH, UCOBEV, UKOCON, ZAJZOZ<br>**C(10)**: JUCSAB, DARVOJ, FEMWAV, NEYLIP, NOVCIK<br>**C^{2}_{2}(10)**: BOLWEE, GESYEL<br>**D(2)**:<br>EXURAQ, FODNES, FODNOC, GESYOV, GLHPRD, KETMIG, KUSXIF, KUXJES, PESYUK, RIXXIG, TUTSOR<br>**D^{2}_{2}(5)**: GESYOV<br>**D^{2}_{2}(10)**: GESYOV		
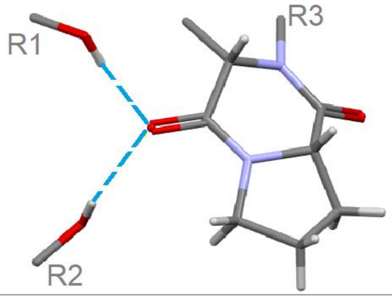	**D12(3)**: PESYUK, UDALUJ		
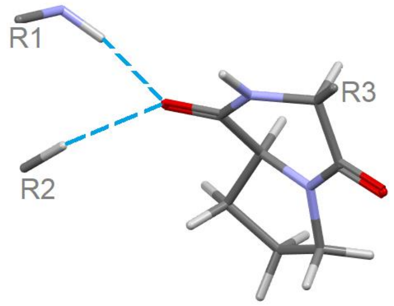	**C^{1}_{2}(5)**: EXURAQ<br>**C^{1}_{2}(6)**: CLPRAL, LPROGL<br>C12(7): NOVCIK, PABRAM<br>**C^{1}_{2}(8)**: CLPRAL, CYPRLE, JEHPUH, YERPIU<br>**C^{1}_{2}(9)**: EBIFAZ, KUXJES, NOVCIK, OSULEU<br>C12(10): OSULEU<br>D12(3): RIXXIG<br>D23(7): KUXJES<br>D23(8): RIMTUE<br>D23(9): KUSFIF	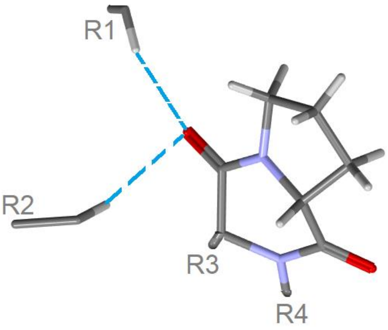	**C12(4)**: LPROGL, XOLQUL<br>**C12(5)**: BAMCEZ, KAQKAD, ODIQUN, ROPVOH, XOLQUL<br>**C^{1}_{2}(6)**: BOLWEE, DARVOJ, JEHPUH, ODIQUN, ROPVOH, XOLQUL<br>**C^{1}_{2}(7)**: DARVID, NOVCIK, YERPIU, XOLQUL<br>**C^{1}_{2}(8)**:

			CLPRAL,<br>CLPRPR,<br>GESYEL,<br>GESYIP,<br>LPROGL,<br>PUYNUS,<br>REFYIK,<br>UKOCON,<br>XOLQUL<br>**$C^{1}_{2}(9)$**:<br>APUHOJ,<br>CLPRPR,<br>DARVID,<br>FODNUI,<br>PABRAM,<br>RIXXIG,<br>XOLQUL<br>**$C^{1}_{2}(10)$**:<br>FODNOC,<br>FODNUI<br>**$D^{1}_{2}(3)$**:<br>GESYOV,<br>RIXXIG<br>**$D^{2}_{3}(8)$**: GLHPRD<br>**$D^{2}_{3}(9)$**: GESYOV<br>**$D^{2}_{3}(10)$**:<br>FODNOC
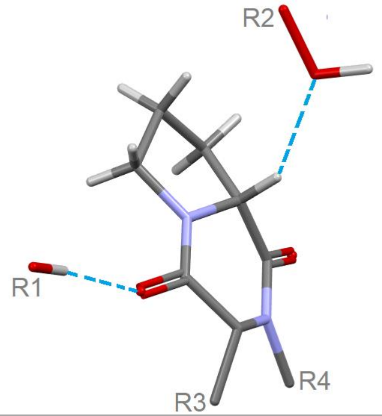	**$C^{2}_{2}(7)$**: BAMCEZ<br>**$C^{2}_{2}(9)$**: FODNOC	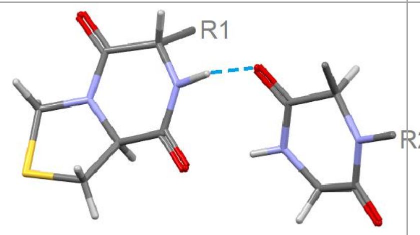	**C(4)**: CLPRAL<br>**$C^{2}_{2}(10)$**:<br>CLPRAL<br>**D(2)**: BOLWEE
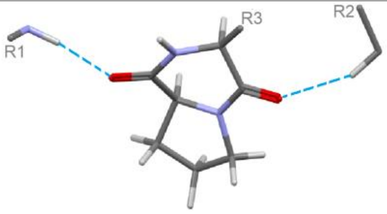	**$C^{2}_{2}(10)$**: CLPRAL, EBIFAZ, LPROGL, MOPQUF, UDALUJ<br>D22(8): RIXXIG	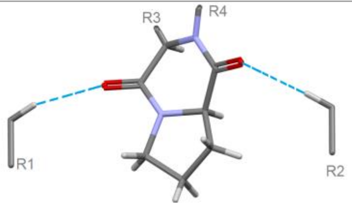	**$C^{2}_{2}(9)$**:<br>APUHOJ,<br>BAMCEZ,<br>CLPRPE,<br>GESYOV,<br>XOLQUL<br>**$C^{2}_{2}(10)$**:<br>CLPRAL,<br>LIKNUO,<br>MOPQUF,<br>XOLQUL<br>**$D^{2}_{2}(8)$**:<br>GESYOV,<br>NOVCIK,<br>RIXXIG,<br>REFYIK,<br>TUTSOR
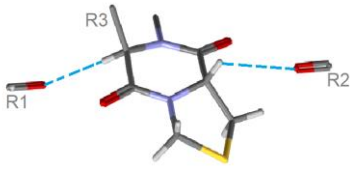	**$D^{2}_{2}(8)$**: GESYOV	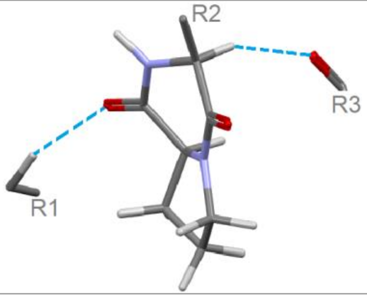	**$C^{2}_{2}(10)$**:<br>CLPRAL,<br>FODNOC<br>**$D^{2}_{2}(7)$**: GESYOV<br>**$D^{2}_{2}(8)$**: GESYOV

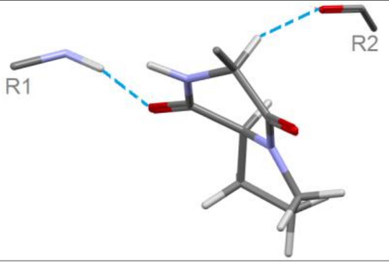	**C^2_2(10)**: CLPRAL, CYPRLE<br>**D^2_2(7)**: GESYOV		
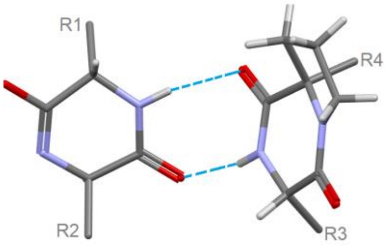	**R^2_2(8)**: AKUWUY, BOLWEE, EBIFAZ, IMIHAP	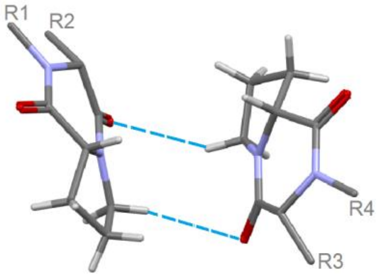	**R^2_2(10)**: BAMCEZ, FODNUI, GESYIP, GESYOV, JAFFEC
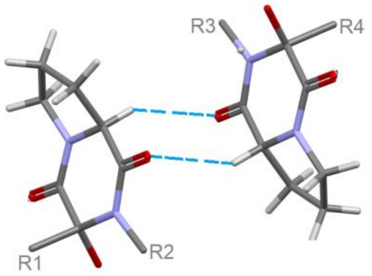	**R^2_2(8)**: JAFFEC	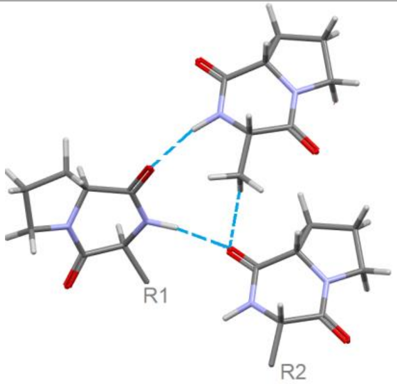	**R^2_3(10)**: CLPRAL
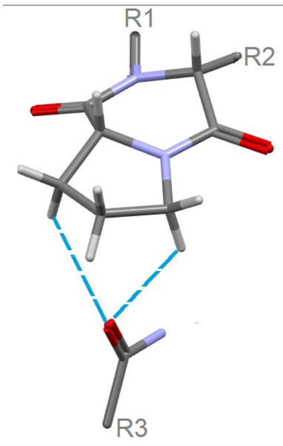	**R^1_2(6)**: APUHOJ, CLPRPR, JEHPUH	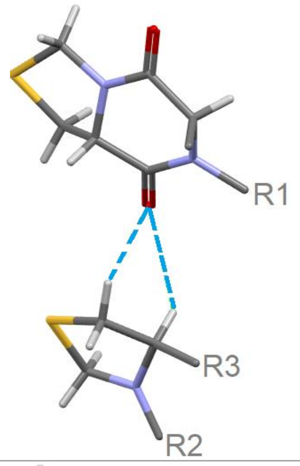	**R^1_2(5)**: GESYOV
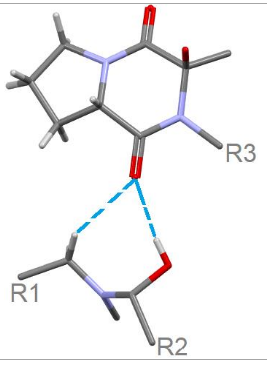	**R^1_2(7)**: FODNOC, GESYOV	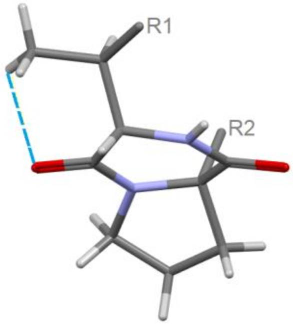	**S(6)**: AKUWUY, FODNUI

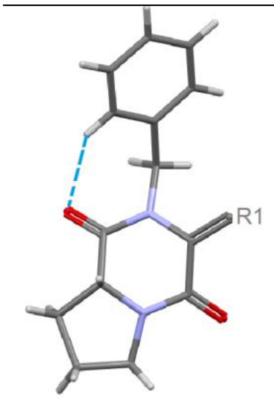	**S(7)**: BUDDOJ, DITGIZ	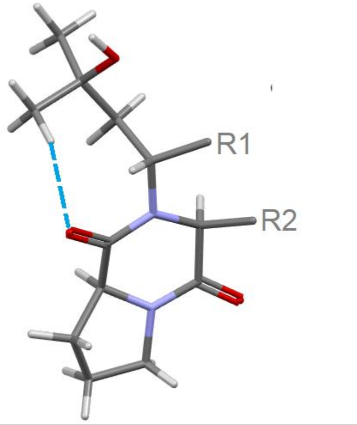	**S(8)**: FEMWAY
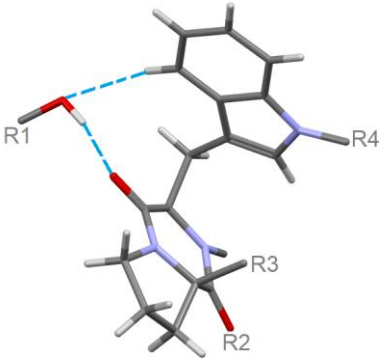	**$R^{2}_{2}(10)$**: BIDDOJ	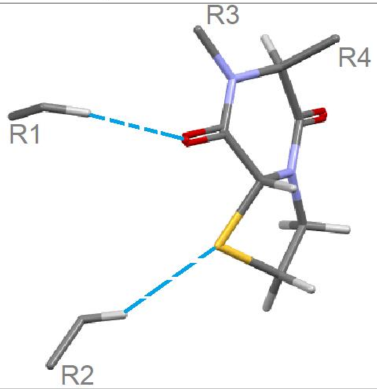	**$C^{2}_{2}(10)$**: GESYEL **$D^{2}_{2}(7)$**: GESYOV
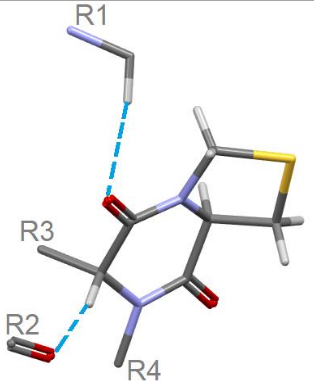	**$D^{2}_{2}(6)$**: GESYOV	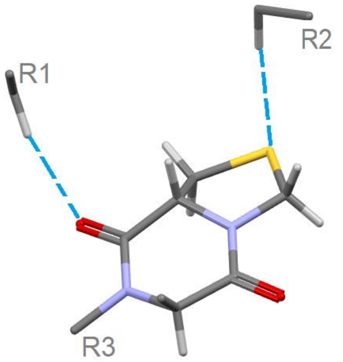	**$D^{2}_{2}(7)$**: GESYOV

Table S3. Library of proline-DKP-based supramolecular synthons (CSD, ver. May 2021, see blue entries in Tb. S1), up to 10-membered H-bonding motifs.

CSD ref. code	*Synthon*	*Interactions forming synthon*
AKUWUY	C(10)	$_{(DKP-NH)}$N-H···O$_{(C=O-DKP)}$
	S(6)	$_{(CH)}$C-H···O=C$_{(DKP)}$
	$R^{2}_{2}(8)$	[$_{(DKP-NH)}$N-H···O=C$_{(DKP)}$]$_2$
APUHOJ	C(5)	$_{(Pro-CH)}$C-H···O=C$_{(DKP)}$
	C(6)	$_{(Pro-CH)}$C-H···O=C$_{(DKP)}$
	$C^{1}_{2}(9)$	[$_{(Pro-CH)}$C-H···O=C$_{(DKP)}$]$_2$
	$C^{2}_{2}(9)$	[$_{(Pro-CH)}$C-H···O=C$_{(DKP)}$]$_2$
	$R^{1}_{2}(6)$	[$_{(CH)}$C-H···O=C$_{(DKP)}$]$_2$
BAMCEZ	C(6)	$_{(OH)}$O-H···O=C$_{(DKP)}$
	C(6)	$_{(Pro-CH)}$C-H···O=C$_{(DKP)}$
	$C^{1}_{2}(5)$	[$_{(Pro-CH)}$C-H···O=C$_{(DKP)}$]$_2$
	$C^{1}_{2}(9)$	$_{(Pro-CH)}$C-H···O=C$_{(DKP)\ \&\ (OH)}$O-H···C-H$_{(CH-Pro)}$
	$C^{2}_{2}(7)$	$_{(OH)}$O-H···O=C$_{(DKP)\ \&\ (OH)}$O-H···C-H$_{(CH-Pro)}$
	$C^{2}_{2}(9)$	[$_{(Pro-CH)}$C-H···O=C$_{(DKP)}$]$_2$
	$C^{2}_{2}(10)$	[$_{(Pro-CH)}$C-H···O=C$_{(DKP)}$]$_2$
	$R^{2}_{2}(10)$	[$_{(Pro-CH)}$C-H···O=C$_{(DKP)}$]$_2$
	$R^{2}_{2}(10)$	[$_{(OH)}$O-H···C-H$_{(CH-DKP)}$]$_2$
BIDDOJ	S(7)	$_{(CH)}$C-H···O=C$_{(DKP)}$
	D(2)	$_{(OH)}$O-H···O=C$_{(DKP)}$
	C(8)	$_{(CH)}$C-H···O=C$_{(DKP)}$

	$R^2_2(10)$	$_{(OH)}$O-H···O=C$_{(DKP)}$ & $_{(CH)}$C-H···O-H$_{(OH)}$
BOLWEE	D(2)	$_{(DKP-NH)}$N-H···O=C$_{(DKP)}$
	D(2)	$_{(Pro-CH)}$C-H···O=C$_{(DKP)}$
	$C^1_2(6)$	[$_{(CH)}$C-H···O=C$_{(DKP)}$]$_2$
	$C^2_2(10)$	$_{(DKP-NH)}$N-H···O=C$_{(DKP)}$ & $_{(CH)}$C-H···O=C$_{(DKP)}$
	$R^2_2(8)$	[$_{(DKP-NH)}$N-H···O=C$_{(DKP)}$]$_2$
CLPRAL	C(4)	$_{(Pro-NH)}$N-H···O=C$_{(DKP)}$
	C(4)	$_{(DKP-CH)}$C-H···O=C$_{(DKP)}$
	C(6)	$_{(CH)}$C-H···O=C$_{(DKP)}$
	$C^1_2(6)$	$_{(DKP-NH)}$N-H···O=C$_{(DKP)}$ & $_{(CH)}$C-H···O=C$_{(DKP)}$
	$C^1_2(8)$	$_{(DKP-NH)}$N-H···O=C$_{(DKP)}$ & $_{(Pro-CH)}$C-H···O=C$_{(DKP)}$
		[$_{DKP-CH)}$C-H···O=C$_{(DKP)}$]$_2$
		$_{(CH)}$C-H···O=C$_{(DKP)}$ & $_{(Pro-CH)}$C-H···O=C$_{(DKP)}$
		$_{(DKP-CH)}$C-H···O=C$_{(DKP)}$ & $_{(Pro-CH)}$C-H···O=C$_{(DKP)}$
	$C^2_2(10)$	$_{(Pro-CH)}$C-H···O=C$_{(DKP)}$ & $_{(DKP-NH)}$N-H···O=C$_{(DKP)}$
		$_{(CH)}$C-H···O=C$_{(DKP)}$ & $_{(DKP-NH)}$N-H···O=C$_{(DKP)}$
		[$_{Pro-CH)}$C-H···O=C$_{(DKP)}$]$_2$
		[$_{DKP-CH)}$C-H···O=C$_{(DKP)}$]$_2$
		[$_{(CH)}$C-H···O=C$_{(DKP)}$]$_2$
	$R^2_2(10)$	$_{(Pro-CH)}$C-H···O=C$_{(DKP)}$ & $_{(CH)}$C-H···O=C$_{(DKP)}$
		[$_{(DKP-NH)}$N-H···O=C$_{(DKP)}$]$_2$
CLPRPR	C(5)	$_{(Pro-CH)}$C-H···O=C$_{(DKP)}$
	C(6)	$_{(Pro-CH)}$C-H···O=C$_{(DKP)}$
	$C^1_2(9)$	[$_{(Pro-CH)}$C-H···O=C$_{(DKP)}$]$_2$
	$C^2_2(9)$	[$_{(Pro-CH)}$C-H···O=C$_{(DKP)}$]$_2$
	$R^1_2(6)$	[$_{(Pro-CH)}$C-H···O=C$_{(DKP)}$]$_2$
CYPRLE	C(5)	$_{(DKP-NH)}$N-H···O=C$_{(DKP)}$
	C(6)	$_{(Pro-CH)}$C-H···O=C$_{(DKP)}$]
	C(7)	$_{(CH)}$C-H···O=C$_{(DKP)}$
	$C^1_2(8)$	$_{(Pro-NH)}$N-H···O=C$_{(DKP)}$ & $_{(CH)}$C-H···O=C$_{(DKP)}$
	$C^2_2(10)$	$_{(NH)}$N-H···O=C$_{(DKP)}$
DARVID	C(8)	$_{(Pro-CH)}$C-H···O=C
		$_{(CH)}$C-H···O=C$_{(DKP)}$
	$C^1_2(7)$	[$_{(CH)}$C-H···O=C$_{(DKP)}$]$_2$
	$C^1_2(9)$	[$_{(CH)}$C-H···O=C$_{(DKP)}$]$_2$
	$C^2_2(8)$	$_{(CH)}$C-H···O=C$_{(DKP)}$ & $_{(Pro-CH)}$C-H···O=C$_{(C-O-C)}$
	$R^2_2(10)$	[$_{(DKP-CH)}$C-H···O=C$_{(DKP)}$]$_2$
DARWOJ	C(4)	$_{(Pro-CH)}$C-H···O=C
		$_{(DKP-CH)}$C-H···O=C$_{(DKP)}$
	C(6)	$_{(DKP-CH)}$C-H···O=C
	C(10)	$_{(CH)}$C-H···O=C$_{(DKP)}$
	$C^1_2(6)$	$_{(Pro-CH)}$C-H···O=C & $_{(DKP-CH)}$C-H···O=C$_{(DKP)}$
	$C^2_2(10)$	$_{(DKP-CH)}$C-H···O=C$_{(DKP)}$ & $_{(Pro-CH)}$C-H···O=C
	$R^2_1(6)$	$_{(DKP-CH)}$C-H···O=C$_{(DKP)}$ & $_{(CH)}$C-H···O=C
DITGIZ	S(7)	$_{(CH)}$C-H···O=C$_{(DKP)}$
	C(9)	$_{(CH)}$C-H···O=C$_{(DKP)}$
DUNVIS	C(5)	$_{(DKP-NH)}$N-H···O=C$_{(DKP)}$
EBIFAZ	C(5)	$_{(DKP-NH)}$N-H···O=C$_{(DKP)}$
	C(5)	$_{(DKP-CH)}$C-H···O=C$_{(DKP)}$
	C(8)	$_{(CH)}$C-H···O=C$_{(DKP)}$
	$C^1_2(9)$	$_{(DKP-NH)}$N-H···O=C$_{(DKP)}$ & $_{(CH)}$C-H···O=C$_{(DKP)}$
	$C^1_2(10)$	$_{(OH)}$O-H···O=C$_{(DKP)}$ & $_{(DKP-CH)}$C-H···O=C$_{(DKP)}$
	$C^2_2(10)$	$_{(DKP-NH)}$N-H···O=C$_{(DKP)}$ & $_{(DKP-CH)}$C-H···O=C$_{(DKP)}$
	$R^2_2(8)$	$_{(DKP-CH)}$C-H···O=C$_{(DKP)}$ & $_{(DKP-NH)}$N-H···O=C$_{(DKP)}$
EXURAZ	D(2)	$_{(NH)}$N-H···O=C$_{(DKP)}$
		$_{(CH)}$C-H···O=C$_{(DKP)}$
	C(6)	$_{(DKP-NH)}$N-H···O=C$_{(DKP)}$
		$_{(NH)}$N-H···O=C$_{(DKP)}$
	$C^1_2(5)$	$_{(NH)}$N-H···O=C$_{(DKP)}$ & $_{(Pro-CH)}$C-H···O=C$_{(DKP)}$
	$D^3_2(9)$	[$_{(NH)}$N-H···O=C$_{(DKP)}$]$_2$
FEMWAV	S(7)	$_{(CH)}$C-H···O=C$_{(DKP)}$
	S(8)	$_{(CH)}$C-H···O=C$_{(DKP)}$
	C(8)	$_{(OH)}$O-H···O=C$_{(DKP)}$
FEXXAH	C(4)	$_{(DKP-CH)}$C-H···O=C$_{(DKP)}$
	C(7)	$_{(CH)}$C-H···O=C$_{(DKP)}$
	$C^2_2(9)$	$_{(CH)}$C-H···O=C$_{(DKP)}$ & $_{(CH)}$C-H···O=C$_{(C=O)}$
FODNES	D(2)	$_{(Pro-CH)}$C-H···O=C$_{(DKP)}$
		$_{(NH)}$N-H···O=C$_{(DKP)}$
	C(9)	$_{(CH)}$C-H···O=C$_{(DKP)}$
FODNOC	D(2)	$_{(OH)}$O-H···O=C$_{(DKP)}$

		$_{(CH)}$C-H···O=C$_{(DKP)}$
	$D^{2}_{3}(10)$	[$_{(CH)}$C-H···O=C$_{(DKP)}$]$_{2}$ $_{\&\ (Pro\text{-}CH)}$C-H···O=C$_{(DKP)}$
	$C(7)$	$_{(CH)}$C-H···O=C$_{(DKP)}$
	$C^{1}_{2}(10)$	[$_{(CH)}$C-H···O=C$_{(DKP)}$]$_{2}$
	$C^{2}_{2}(9)$	$_{(OH)}$O-H···O=C$_{(DKP)\ \&\ (DKP\text{-}CH)}$C-H···O=C$_{(DKP)}$
	$C^{2}_{2}(10)$	$_{(Pro\text{-}CH)}$C-H···O=C$_{(DKP)\ \&\ (CH)}$C-H···O=C$_{(DKP)}$
		[$_{(Pro\text{-}CH)}$C-H···O=C$_{(DKP)}$]$_{2}$
	$R^{1}_{2}(7)$	$_{(OH)}$O-H···O=C$_{(DKP)\ \&\ (CH)}$C-H···O=C$_{(DKP)}$
	$R^{2}_{2}(8)$	$_{(CH)}$C-H···O=C$_{(DKP)\ \&\ (CH)}$C-H···O$_{(C\text{-}O\text{-}C)}$
	$R^{2}_{2}(10)$	$_{(OH)}$O-H···O=C$_{(DKP)\ \&\ (CH)}$C-H···O-H$_{(OH)}$
		$_{(OH)}$O-H···O=C$_{(DKP)\ \&\ (CH)}$C-H···O-H$_{(OH)}$
FODNUI	$S(6)$	$_{(CH)}$C-H···O=C$_{(DKP)}$
	$C(6)$	$_{(CH)}$C-H···O=C$_{(DKP)}$
	$C(7)$	$_{(CH)}$C-H···O=C$_{(DKP)}$
	$C(8)$	$_{(CH)}$C-H···O=C$_{(DKP)}$
	$C^{1}_{2}(9)$	$_{(CH)}$C-H···O=C$_{(DKP)\ \&\ (Pro\text{-}CH)}$C-H···O=C$_{(DKP)}$
	$C^{1}_{2}(10)$	$_{(CH)}$C-H···O=C$_{(DKP)\ \&\ (Pro\text{-}CH)}$C-H···O=C$_{(DKP)}$
	$R^{2}_{2}(10)$	[$_{(Pro\text{-}CH)}$C-H···O=C$_{(DKP)}$]$_{2}$
GESYAH	$C(5)$	$_{(CH)}$C-H···O=C$_{(DKP)}$
	$C(6)$	$_{(CH)}$C-H···O=C$_{(DKP)}$
GESYEI	$C(5)$	$_{(DKP\text{-}CH)}$C-H···O=C$_{(DKP)}$
	$C(6)$	$_{(Pro\text{-}CH)}$C-H···O=C$_{(DKP)}$
		$_{(CH)}$C-H···O=C$_{(DKP)}$
	$C(9)$	$_{(CH)}$C-H···O=C$_{(DKP)}$
		$_{(CH)}$C-H···S$_{(Pro)}$
	$C^{1}_{2}(8)$	$_{(CH)}$C-H···O=C$_{(DKP)\ \&\ (Pro\text{-}CH)}$C-H···O=C$_{(DKP)}$
	$C^{2}_{2}(10)$	$_{(DKP\text{-}CH)}$C-H···O=C$_{(DKP)\ \&\ (CH)}$C-H···S$_{(Pro)}$
		$_{(DKP)}$C-H···O=C$_{(DKP)\ \&\ (CH)}$C-H···S$_{(Pro)}$
	$R^{2}_{2}(9)$	$_{(DKP\text{-}CH)}$C-H···O=C$_{(DKP)\ \&\ (CH)}$C-H···O=C$_{(DKP)}$
GESYIP	$C(5)$	$_{(Pro\text{-}CH)}$C-H···O=C$_{(DKP)}$
		$_{(CH)}$C-H···O=C$_{(DKP)}$
	$C(9)$	$_{(CH)}$C-H···O=C$_{(DKP)}$
	$C^{1}_{2}(8)$	$_{(CH)}$C-H···O=C$_{(DKP)\ \&\ (Pro\text{-}CH)}$C-H···O=C$_{(DKP)}$
	$R^{2}_{2}(10)$	$_{(Pro\text{-}CH)}$C-H···O=C$_{(DKP)\ \&\ (CH)}$C-H···O=C$_{(DKP)}$
GESYOV	$D(2)$	$_{(DKP\text{-}CH)}$C-H···O=C$_{(DKP)}$
		$_{(Pro\text{-}CH)}$C-H···O=C$_{(DKP)}$
		$_{(Pro\text{-}CH)}$C-H···O=C$_{(DKP)}$
	$D^{1}_{2}(3)$	$_{(Pro\text{-}CH)}$C-H···O=C$_{(DKP)\ \&\ (DKP\text{-}CH)}$C-H···O=C$_{(DKP)}$
	$D^{2}_{2}(5)$	$_{(Pro\text{-}CH)}$C-H···S$_{(Pro)\ \&\ (Pro\text{-}CH)}$C-H···O=C$_{(DKP)}$
		$_{(Pro\text{-}CH)}$C-H···O=C$_{(DKP)\ \&\ (DKP/Pro\text{-}CH)}$C-H···S$_{(Pro)}$
	$D^{2}_{2}(6)$	$_{(CH)}$C-H···O=C$_{(DKP)\ \&\ (DKP\text{-}CH)}$C-H···O=C$_{(DKP)}$
		$_{(Pro\text{-}CH)}$C-H···S$_{(Pro)\ \&\ (DKP/Pro\text{-}CH)}$C-H···O=C$_{(DKP)}$
	$D^{2}_{2}(7)$	$_{(DKP\text{-}CH)}$C-H···S$_{(Pro)\ \&\ (DKP\text{-}CH)}$C-H···O=C$_{(DKP)}$
		$_{(DKP/Pro\text{-}CH)}$C-H···O=C$_{(DKP)\ \&\ (CH\text{-}Pro/DKP)}$C-H···S$_{(Pro)}$
		$_{(DKP\text{-}CH)}$C-H···O=C$_{(DKP)\ \&\ (CH)}$C-H···O=C$_{(DKP)}$
	$D^{2}_{2}(8)$	[$_{(DKP\text{-}CH)}$C-H···O=C$_{(DKP)}$]$_{2}$
		$_{(Pro\text{-}CH)}$C-H···O=C$_{(DKP)}$]$_{2}$
		$_{(Pro\text{-}CH)}$C-H···O=C$_{(DKP)\ \&\ (DKP\text{-}CH)}$C-H···O=C$_{(DKP)}$
		$_{(DKP\text{-}CH)}$C-H···O=C$_{(DKP)\ \&\ (Pro\text{-}CH)}$C-H···O=C$_{(DKP)}$
	$D^{2}_{2}(9)$	$_{(CH)}$C-H···O=C$_{(DKP)\ \&\ (Pro\text{-}CH)}$C-H···S$_{(Pro)}$
	$D^{2}_{3}(9)$	[$_{(CH)}$C-H···O=C$_{(DKP)}$]$_{2}$ $_{\&\ (CH\text{-}Pro)}$C-H···O=C$_{(DKP)}$
	$D^{2}_{2}(10)$	$_{(Pro\text{-}CH)}$C-H···O=C$_{(DKP)\ \&\ (CH)}$C-H···O=C$_{(DKP)}$
		$_{(DKP\text{-}CH)}$C-H···O=C$_{(DKP)\ \&\ (CH)}$C-H···S$_{(Pro)}$
		$_{(DKP\text{-}CH)}$C-H···O=C$_{(DKP)\ \&\ (CH)}$C-H···S$_{(Pro)}$
	$C(9)$	$_{(Pro\text{-}CH)}$C-H···S$_{(Pro)}$
	$C^{1}_{2}(8)$	$_{(DKP\text{-}CH)}$C-H···S$_{(Pro)\ \&\ (Pro\text{-}CH)}$C-H···S$_{(Pro)}$
	$C^{2}_{2}(9)$	$_{(Pro\text{-}CH)}$C-H···O=C$_{(DKP)\ \&\ (Pro\text{-}CH)}$C-H···S$_{(Pro)}$
		[$_{(CH)}$C-H···O=C$_{(DKP)}$]$_{2}$
	$C^{2}_{2}(10)$	$_{(Pro\text{-}CH)}$C-H···O=C$_{(DKP)\ \&\ (Pro\text{-}CH)}$C-H···O=C$_{(DKP)}$
		$_{(Pro\text{-}CH)}$C-H···O=C$_{(DKP)\ \&\ (Pro\text{-}CH)}$C-H···S$_{(Pro)}$
	$R^{1}_{2}(5)$	$_{(Pro\text{-}CH)}$C-H···O=C$_{(DKP)\ \&\ (Pro\text{-}CH)}$C-H···O=C$_{(DKP)}$
	$R^{1}_{2}(7)$	[$_{(CH)}$C-H···O=C$_{(DKP)}$]$_{2}$
	$R^{1}_{2}(9)$	[$_{(CH)}$C-H···O=C$_{(DKP)}$]$_{2}$
	$R^{2}_{2}(7)$	$_{(Pro\text{-}CH)}$C-H···O=C$_{(DKP)\ \&\ (DKP\text{-}CH)}$C-H···S$_{(Pro)}$
	$R^{2}_{2}(9)$	$_{(Pro/DKP\text{-}CH)}$C-H···O=C$_{(DKP)\ \&\ (CH)}$C-H···O=C$_{(DKP)}$
	$R^{2}_{2}(10)$	$_{(CH)}$C-H···O=C$_{(DKP)\ \&\ (CH\text{-}Pro)}$C-H···O=C$_{(DKP)}$
GLHPRD	$D(2)$	$_{(Pro/DKP\text{-}CH)}$C-H···O=C$_{(DKP)}$
		$_{(Pro\text{-}CH)}$C-H···O$_{(H2O)}$
		$_{(Pro\text{-}CH)}$C-H···O=C$_{(DKP)}$
	$D^{1}_{2}(3)$	[$_{(Pro\text{-}CH)}$C-H···O$_{(H2O)}$]$_{2}$

	$D^2_2(5)$	$_{(Pro-CH)}$C-H···O=C$_{(DKP)}$ & $_{(Pro-CH)}$C-H···O$_{(H2O)}$
	$D^2_2(6)$	$_{(Pro-CH)}$C-H···O=C$_{(Pro)}$ & $_{(Pro-CH)}$C-H···O$_{(H2O)}$
		$_{(Pro-CH)}$C-H···O$_{(C=O)}$ & $_{(Pro-CH)}$C-H···O$_{(H2O)}$
	$D^2_2(7)$	$_{(DKP/Pro-CH)}$C-H···O=C$_{(DKP)}$ & $_{(Pro-CH)}$C-H···O$_{(H2O)}$
	$D^2_2(8)$	$_{(Pro-CH)}$C-H···O=C$_{(DKP)}$ & $_{(Pro-CH)}$C-H···O$_{(H2O)}$
	$D^2_2(9)$	$_{(DKP/Pro-CH)}$C-H···O=C$_{(DKP)}$ & $_{(Pro-CH)}$C-H···O$_{(H2O)}$
	$D^2_3(8)$	$_{(Pro/DKP-CH)}$C-H···O=C$_{(DKP)}$ & $_{(Pro-CH)}$C-H···O=C$_{(DKP)}$ & $_{(DKP-CH)}$C-H···O=C$_{(DKP)}$
	$C^2_2(9)$	[$_{(DKP-CH)}$C-H···O=C$_{(DKP)}$]$_2$
	$C^2_2(10)$	$_{(Pro-CH)}$C-H···O=C$_{(DKP)}$ & $_{(Pro-CH)}$C-H···O$_{(C=O)}$
GUGXOW	C(6)	$_{(Pro-CH)}$C-H···O$_{(C=O)}$
		$_{(DKP/Pro-CH)}$C-H···O$_{(C-O-C)}$
	C(9)	$_{(Pro-CH)}$C-H···O$_{(C=O)}$
	$C^2_2(10)$	$_{(Pro-CH)}$C-H···O=C$_{(DKP)}$ & $_{(Pro-CH)}$C-H···O$_{(C=O)}$
	$R^2_2(8)$	$_{(Pro-CH)}$C-H···O=C$_{(DKP)}$ & $_{(DKP-CH)}$C-H···O$_{(C-O-C)}$
IMIHAP	D(2)	$_{(DKP-CH)}$C-H···O=C$_{(DKP)}$
	$D^2_3(7)$	$_{[(CH)}$C-H···O=C$_{(DKP)]2}$ & $_{(DKP-NH)}$N-H···O=C$_{(DKP)}$
	C(9)	$_{(CH)}$C-H···O=C$_{(DKP)}$
	C(10)	$_{(OH)}$O-H···O=C$_{(DKP)}$
	$C^1_2(9)$	$_{(CH)}$C-H···O=C$_{(DKP)}$ & $_{(DKP/NH)}$N-H···O=C$_{(DKP)}$
	$R^2_2(8)$	[$_{(DKP-NH)}$N-H···O=C$_{(DKP)}$]$_2$
JAFFEC	C(7)	$_{(CH)}$C-H···O=C$_{(DKP)}$
	$R^2_2(8)$	[$_{(Pro/DKP-CH)}$C-H···O=C$_{(DKP)}$]$_2$
	$R^2_2(10)$	[$_{(Pro/DKP-CH)}$C-H···O=C$_{(DKP)}$]$_2$
JEHPUH	C(5)	$_{(DKP-NH)}$N-H···O=C$_{(DKP)}$
		$_{(CH)}$C-H···O=C$_{(DKP)}$
	C(10)	$_{(NH)}$N-H···O=C$_{(DKP)}$
	$C^1_2(8)$	$_{(CH)}$C-H···O=C$_{(DKP)}$ & $_{(NH)}$N-H···O=C$_{(DKP)}$
	$C^2_2(8)$	$_{(DKP/Pro-CH)}$C-H···O=C$_{(DKP)}$ & $_{(NH)}$N-H···O=C$_{(DKP)}$
	$C^2_2(10)$	$_{(Pro-CH)}$C-H···O=C$_{(DKP)}$ & $_{(DKP/NH)}$N-H···O=C$_{(DKP)}$
	$R^1_2(6)$	$_{(CH)}$C-H···O=C$_{(DKP)}$ & $_{(DKP-NH)}$N-H···O=C$_{(DKP)}$
JIFNER	C(6)	$_{(OH)}$O-H···O=C$_{(DKP)}$
		$_{(Pro-CH)}$C-H···O=C$_{(DKP)}$
	C(7)	$_{(CH)}$C-H···O=C$_{(DKP)}$
	$C^1_2(8)$	$_{(Pro-CH)}$C-H···O=C$_{(DKP)}$ & $_{(OH)}$O-H···O=C$_{(DKP)}$
	$C^2_2(9)$	$_{(CH)}$C-H···O=C$_{(DKP)}$ & $_{(CH)}$C-H···O$_{(OH)}$
	$C^2_2(10)$	$_{(CH)}$C-H···O$_{(OH)}$ & $_{(OH)}$O-H···O=C$_{(DKP)}$
JUCSAB	C(5)	$_{(DKP-CH)}$C-H···O=C$_{(DKP)}$
	C(6)	$_{(CH)}$C-H···O=C$_{(DKP)}$
	C(10)	$_{(CH)}$C-H···O=C$_{(DKP)}$
KAQXAD	S(6)	$_{(CH)}$C-H···O=C$_{(DKP)}$
	C(6)	$_{(Pro-CH)}$C-H···O=C$_{(DKP)}$
	$C^1_2(5)$	[$_{(Pro-CH)}$C-H···O=C$_{(DKP)}$]$_2$
KETMIG	D(2)	$_{(Pro-CH)}$C-H···O=C$_{(DKP)}$
	C(5)	$_{(Pro-CH)}$C-H···O=C$_{(DKP)}$
	C(8)	$_{(CH)}$C-H···O=C$_{(DKP)}$
	C(9)	$_{(CH)}$C-H···O=C$_{(DKP)}$
	$R^1_2(7)$	[$_{(CH)}$C-H···O=C$_{(DKP)}$]$_2$
KUSXIF	D(2)	$_{(DKP-NH)}$N-H···O=C$_{(DKP)}$
		$_{(CH)}$C-H···O=C$_{(DKP)}$
	$D^2_3(9)$	$_{(CH)}$C-H···O=C$_{(DKP)}$ & $_{(NH)}$N-H···O=C$_{(DKP)}$
		$_{(CH)}$C-H···O=C$_{(DKP)}$ & $_{(DKP-NH)}$N-H···O=C$_{(DKP)}$
	C(6)	$_{(CH)}$C-H···O=C$_{(DKP)}$
	C(9)	$_{(CH)}$C-H···O=C$_{(DKP)}$
	S(7)	$_{(CH)}$C-H···O=C$_{(DKP)}$
	$R^2_2(8)$	[$_{(DKP-NH)}$N-H···O=C$_{(DKP)}$]$_2$
KUXJES	D(2)	$_{(Pro-CH)}$C-H···O=C$_{(DKP)}$
	$D^2_3(7)$	$_{(DKP-CH)}$C-H···O=C$_{(DKP)}$ & $_{(CH)}$C-H···O=C$_{(DKP)}$ & $_{(NH)}$N-H···O=C$_{(DKP)}$
	C(8)	$_{(Pro-CH)}$C-H···O=C$_{(DKP)}$
	C(9)	$_{(CH)}$C-H···O=C$_{(DKP)}$
	C(10)	$_{(OH)}$O-H···O=C$_{(DKP)}$
	$C^1_2(9)$	$_{(DKP-NH)}$N-H···O=C$_{(DKP)}$ & $_{(CH)}$C-H···O=C$_{(DKP)}$
	$R^1_2(6)$	$_{(CH)}$C-H···O=C$_{(DKP)}$ & $_{(OH)}$O-H···O=C$_{(DKP)}$
	$R^2_2(8)$	[$_{(NH)}$N-H···O=C$_{(DKP)}$]$_2$
LEHREW	C(6)	$_{(CH)}$C-H···O=C$_{(DKP)}$
	C(8)	$_{(NH)}$N-H···O=C$_{(DKP)}$
LIKNUO	C(7)	$_{(Pro-CH)}$C-H···O=C$_{(DKP)}$
	C(10)	$_{(CH)}$C-H···O$_{(C=O)}$
	$C^2_2(10)$	$_{(DKP-CH)}$C-H···O$_{(C=O)}$ & $_{(Pro-CH)}$C-H···O=C$_{(DKP)}$
		[$_{(Pro-CH)}$C-H···O=C$_{(DKP)}$]$_2$

LPROGL	C(5)	$_{\text{(DKP/Pro-CH)}}$C-H···O=C$_{\text{(DKP)}}$
		$_{\text{(DKP-NH)}}$N-H···O=C$_{\text{(DKP)}}$
	C(6)	$_{\text{(Pro-CH)}}$C-H···O=C$_{\text{(DKP)}}$
	$C^1_2(4)$	$_{\text{(DKP-CH)}}$C-H···O=C$_{\text{(DKP) \& (DKP-CH)}}$C-H···O=C$_{\text{(DKP)}}$
	$C^1_2(6)$	$_{\text{(DKP-NH)}}$N-H···O=C$_{\text{(DKP) \& (Pro-CH)}}$C-H···O=C$_{\text{(DKP)}}$
	$C^1_2(8)$	[$_{\text{(Pro-CH)}}$C-H···O=C$_{\text{(DKP)}}$]$_2$
	$C^2_2(8)$	$_{\text{(DKP-CH)}}$C-H···O=C$_{\text{(DKP) \& (DKP/Pro-CH)}}$C-H···O=C$_{\text{(DKP)}}$
		$_{\text{(DKP-NH)}}$N-H···O=C$_{\text{(DKP) \& (DKP-CH)}}$C-H···O=C$_{\text{(DKP)}}$
	$C^2_2(10)$	$_{\text{(DKP-CH)}}$C-H···O=C$_{\text{(DKP) \& (DKP/Pro-CH)}}$C-H···O=C$_{\text{(DKP)}}$
		$_{\text{(DKP-NH)}}$N-H···O=C$_{\text{(DKP) \& (DKP/Pro-CH)}}$C-H···O=C$_{\text{(DKP)}}$
		$_{\text{(DKP-NH)}}$N-H···O=C$_{\text{(DKP) \& (DKP-CH)}}$C-H···O=C$_{\text{(DKP)}}$
	$R^2_2(8)$	$_{\text{(DKP-CH)}}$C-H···O=C$_{\text{(DKP) \& (Pro-CH)}}$C-H···O=C$_{\text{(DKP)}}$
		[$_{\text{(Pro-NH)}}$N-H···O=C$_{\text{(DKP)}}$]$_2$
MOPQUF	C(7)	$_{\text{(NH)}}$N-H···O=C$_{\text{(DKP)}}$
		$_{\text{(CH)}}$C-H···O=C$_{\text{(DKP)}}$
	C(9)	$_{\text{(CH)}}$C-H···O=C$_{\text{(DKP)}}$
	$R^1_2(8)$	$_{\text{(NH)}}$N-H···O=C$_{\text{(DKP) \& (CH)}}$C-H···O=C$_{\text{(DKP)}}$
	$C^2_2(10)$	[$_{\text{(CH)}}$C-H···O=C$_{\text{(DKP)}}$]$_2$
NEYLIP	S(7)	$_{\text{(DKP-NH)}}$N-H···O=C$_{\text{(DKP)}}$
	C(8)	$_{\text{(Pro-NH)}}$N-H···O=C$_{\text{(DKP)}}$
	C(10)	$_{\text{(CH)}}$C-H···O=C$_{\text{(DKP)}}$
	$C^2_2(8)$	$_{\text{(OH)}}$O-H···O=C$_{\text{(DKP) \& (Pro-CH)}}$C-H···O-H$_{\text{(OH)}}$
	$C^2_2(10)$	$_{\text{(CH)}}$C-H···O=C$_{\text{(DKP) \& (Pro-CH)}}$C-H···O=H$_{\text{(OH)}}$
	$R^1_2(6)$	$_{\text{(OH)}}$O-H···O=C$_{\text{(DKP)}}$
NOVCIK	C(6)	$_{\text{(DKP-CH)}}$C-H···O=C$_{\text{(C=O-Pro)}}$
	C(7)	$_{\text{(Pro-CH)}}$C-H···O=C$_{\text{(DKP)}}$
	C(9)	$_{\text{(DKP-CH)}}$C-H···O=C$_{\text{(C=O)}}$
	C(10)	$_{\text{(DKP-CH)}}$C-H···O=C$_{\text{(DKP)}}$
		$_{\text{(Pro-CH)}}$C-H···S$_{\text{(Pro)}}$
	$C^1_2(7)$	$_{\text{(NH)}}$N-H···O=C$_{\text{(DKP) \& (Pro-CH)}}$C-H···O=C$_{\text{(DKP)}}$
		[$_{\text{(Pro-CH)}}$C-H···O=C$_{\text{(DKP)}}$]$_2$
	$C^1_2(9)$	$_{\text{(NH)}}$N-H···O=C$_{\text{(DKP)}}$
		$_{\text{(Pro-CH)}}$C-H···O=C$_{\text{(DKP)}}$
	$C^2_2(9)$	$_{\text{(DKP-CH)}}$C-H···O=C$_{\text{(c=o) \& (Pro-CH)}}$C-H···O=C$_{\text{(DKP)}}$
		$_{\text{(Pro-CH)}}$C-H···S$_{\text{(Pro) \& (Pro-CH)}}$C-H···O=C$_{\text{(DKP)}}$
	$C^2_2(10)$	$_{\text{(CH)}}$C-H···O=C$_{\text{(C=O-Pro) \& (Pro-CH)}}$C-H···O=C$_{\text{(DKP)}}$
		$_{\text{(DKP-CH)}}$C-H···O=C$_{\text{(C=O-Pro) \& (Pro-CH)}}$C-H···O=C$_{\text{(DKP)}}$
	$R^2_2(8)$	$_{\text{(NH)}}$N-H···O=C$_{\text{(DKP) \& (Pro-CH)}}$C-H···O=C$_{\text{(DKP)}}$
		$_{\text{(Pro-CH)}}$C-H···S$_{\text{(Pro) \& (Pro-CH)}}$C-H···O=C$_{\text{(DKP)}}$
ODIQUN	C(4)	$_{\text{(DKP/Pro-CH)}}$C-H···O=C$_{\text{(DKP)}}$
	C(6)	$_{\text{(Pro-CH)}}$C-H···O=C$_{\text{(DKP)}}$
	C(8)	$_{\text{(DKP/Pro-CH)}}$C-H···O=C$_{\text{(DKP)}}$
	$C^1_2(5)$	[$_{\text{(Pro-CH)}}$C-H···O=C$_{\text{(DKP)}}$]$_2$
	$C^1_2(6)$	$_{\text{(DKP-CH)}}$C-H···O=C$_{\text{(C=O-Pro) \& (Pro-CH)}}$C-H···O=C$_{\text{(DKP)}}$
	$C^2_2(10)$	[$_{\text{(Pro-CH)}}$C-H···O=C$_{\text{(DKP)}}$]$_2$
		$_{\text{(Pro/DKP-CH)}}$C-H···O=C$_{\text{(DKP) \& (Pro-CH)}}$C-H···O=C$_{\text{(DKP)}}$
	$R^1_2(6)$	[$_{\text{(Pro/DKP-CH)}}$C-H···O=C$_{\text{(DKP)}}$]$_2$
	$R^2_2(10)$	[$_{\text{(Pro-CH)}}$C-H···O=C$_{\text{(DKP)}}$]$_2$
	$R^2_3(10)$	[$_{\text{(DKP-CH)}}$C-H···O=C$_{\text{(DKP)}}$]$_3$
OSULEU	C(6)	$_{\text{(Pro-CH)}}$C-H···O=C$_{\text{(DKP)}}$
	C(8)	$_{\text{(Pro-NH)}}$N-H···O=C$_{\text{(DKP)}}$
	C(9)	$_{\text{(CH)}}$C-H···O=C$_{\text{(DKP)}}$
	C(10)	$_{\text{(Pro-CH)}}$C-H···O=C$_{\text{(DKP)}}$
	$C^1_2(9)$	$_{\text{(NH)}}$N-H···O=C$_{\text{(DKP) \& (CH)}}$C-H···O=C$_{\text{(DKP)}}$
	$C^1_2(10)$	$_{\text{(Pro-NH)}}$N-H···O=C$_{\text{(DKP) \& (CH)}}$C-H···O=C$_{\text{(DKP)}}$
	$C^2_2(10)$	$_{\text{(Pro-NH)}}$N-H···O=C$_{\text{(DKP) \& (Pro-CH)}}$C-H···O=C$_{\text{(DKP)}}$
PABRAM	C(4)	$_{\text{(DKP-NH)}}$N-H···O=C$_{\text{(DKP)}}$
	C(5)	$_{\text{(Pro-CH)}}$C-H···O=C$_{\text{(DKP)}}$
	C(6)	$_{\text{(CH)}}$C-H···O=C$_{\text{(DKP)}}$
	$C^1_2(7)$	$_{\text{(DKP-NH)}}$N-H···O=C$_{\text{(DKP) \& (Pro-CH)}}$C-H···O=C$_{\text{(DKP)}}$
	$C^1_2(9)$	$_{\text{(CH)}}$C-H···O=C$_{\text{(DKP) \& (Pro-CH)}}$C-H···O=C$_{\text{(DKP)}}$
	$C^2_2(10)$	$_{\text{(DKP-NH)}}$N-H···O=C$_{\text{(DKP) \& (CH)}}$C-H···O=C$_{\text{(DKP)}}$
	$R^1_2(6)$	$_{\text{(DKP-NH)}}$N-H···O=C$_{\text{(DKP) \& (CH)}}$C-H···O=C$_{\text{(DKP)}}$
	$R^2_2(9)$	$_{\text{(DKP-NH)}}$N-H···O=C$_{\text{(DKP) \& (Pro-CH)}}$C-H···O=C$_{\text{(DKP)}}$
PESYUK	D(2)	$_{\text{(OH)}}$O-H···O=C$_{\text{(DKP)}}$
		$_{\text{(Pro-CH)}}$C-H···O=C$_{\text{(DKP)}}$
	$D^1_2(3)$	[$_{\text{(OH)}}$O-H···O=C$_{\text{(DKP)}}$]$_2$
	$D^2_2(4)$	$_{\text{(OH)}}$O-H···O=C$_{\text{(DKP) \& (Pro-CH)}}$C-H···O-H$_{\text{(OH)}}$
	$D^2_2(7)$	$_{\text{(OH)}}$O-H···O=C$_{\text{(DKP) \& (Pro-CH)}}$C-H···O-H$_{\text{(OH)}}$
		$_{\text{(Pro-CH)}}$C-H···O-H$_{\text{(OH) \& (Pro-CH)}}$C-H···O=C $_{\text{(DKP)}}$

		$_{(CH)}$C-H···O=C$_{(DKP)}$ & $_{(Pro-CH)}$C-H···O-H$_{(OH)}$
	$D^2_2(8)$	$_{(OH)}$O-H···O=C$_{(DKP)}$ & $_{(Pro-CH)}$C-H···O=C$_{(DKP)}$
		$_{(OH)}$O-H···O=C$_{(DKP)}$ & $_{(Pro-CH)}$C-H···O-H$_{(OH)}$
		$_{(OH)}$O-H···O=C$_{(DKP)}$ & $_{(Pro-CH)}$C-H···O=C$_{(DKP)}$
	$D^2_2(9)$	$_{(OH)}$O-H···O=C$_{(DKP)}$ & $_{(Pro-CH)}$C-H···O-H$_{(OH)}$
	$D^2_2(10)$	[$_{(OH)}$O-H···O=C$_{(DKP)}$]$_2$
		[$_{(OH)}$O-H···O=C$_{(DKP)}$]$_2$
		$_{(OH)}$O-H···O=C$_{(DKP)}$ & $_{(Pro-CH)}$C-H···O=C $_{(DKP)}$
		$_{(Pro-CH)}$C-H···O-H$_{(OH)}$ & $_{(Pro-CH)}$C-H···O=C $_{(DKP)}$
	$C^2_2(10)$	[$_{(Pro-CH)}$C-H···O=C$_{(DKP)}$]$_2$
	$R^2_2(7)$	$_{(OH)}$O-H···O=C$_{(DKP)}$ & $_{(Pro-CH)}$C-H···O-H$_{(OH)}$
PUYNUS	C(5)	$_{(Pro-CH)}$C-H···O=C$_{(DKP)}$
	C(6)	$_{(DKP/Pro-CH)}$C-H···O=C$_{(DKP)}$
	C(7)	$_{(CH)}$C-H···O=C$_{(DKP)}$
	C(8)	$_{(DKP/Pro-CH)}$C-H···O=C$_{(DKP)}$
	$C^1_2(8)$	[$_{(DKP/-CH)}$C-H···O=C$_{(DKP)}$]$_2$
	$C^2_2(10)$	[$_{(Pro-CH)}$C-H···O=C$_{(DKP)}$]$_2$
QAXRUT	C(4)	$_{(DKP-CH)}$C-H···O=C$_{(DKP)}$
	C(6)	$_{(CH)}$C-H···O=C$_{(DKP)}$
	C(8)	$_{(CH)}$C-H···O=C$_{(DKP)}$
	$C^2_2(10)$	$_{(CH)}$C-H···O=C$_{(DKP)}$ & $_{(DKP-CH)}$C-H···O=C$_{(DKP)}$
	$R^1_2(6)$	[$_{(CH)}$C-H···O=C$_{(DKP)}$]$_2$
QOJXIY	S(6)	$_{(OH)}$O-H···O=C$_{(DKP)}$
	$R^1_2(6)$	[$_{(CH)}$C-H···O=C$_{(DKP)}$]$_2$
REFYIK	C(6)	$_{(Pro-CH)}$C-H···O=C$_{(DKP)}$
	C(7)	$_{(DKP-CH)}$C-H···O=C$_{(C=O)}$
	C(9)	$_{(Pro-CH)}$C-H···O=C$_{(DKP)}$
	$C^1_2(8)$	$_{(DKP-CH)}$C-H···O=C$_{(C=O)}$ & $_{(DKP-CH)}$C-H···O=C$_{(C=O)}$
	$C^2_2(9)$	$_{(CH)}$C-H···O=C$_{(DKP)}$ & $_{(CH)}$C-H···O=C$_{(C=O)}$
	$C^2_2(10)$	$_{(CH)}$C-H···O=C$_{(DKP)}$ & $_{(DKP-CH)}$C-H···O=C$_{(C=O)}$
RIMTUE	D(2)	$_{(DKP-NH)}$N-H···O=C$_{(DKP)}$
	C(5)	$_{(DKP-CH)}$C-H···O=C$_{(DKP)}$
	$R^1_2(6)$	[$_{(CH)}$C-H···O=C$_{(DKP)}$]$_2$
	$R^2_2(8)$	[$_{(DKP-NH)}$N-H···O=C$_{(DKP)}$]$_2$
	$D^2_3(8)$	$_{(DKP)}$N-H···O=C$_{(DKP)}$ & $_{(DKP-CH)}$C-H···O=C$_{(C=O)}$
	$D^3_3(10)$	$_{(NH)}$N-H···O=C$_{(DKP)}$ & $_{(DKP-CH)}$C-H···O=C$_{(DKP)}$
RISDIH	S(6)	$_{(OH)}$O-H···O=C$_{(DKP)}$
	C(5)	$_{(OH)}$O-H···O=C$_{(DKP)}$
	$C^2_2(10)$	$_{(CH)}$C-H···O-H$_{(OH)}$ & $_{(CH)}$C-H···O=C$_{(DKP)}$
RIXXIG	D(2)	$_{(DKP)}$N-H···O=C$_{(DKP)}$
		$_{(Pro-CH)}$C-H···O=C$_{(DKP)}$
	C(9)	$_{(DKP)}$N-H···O=C$_{(DKP)}$
		$_{(CH)}$C-H···O=C$_{(DKP)}$
	$C^1_2(9)$	[$_{(CH)}$C-H···O=C$_{(DKP)}$]$_2$
	$D^1_2(3)$	$_{(DKP-NH)}$N-H···O=C$_{(DKP)}$ & $_{(Pro-CH)}$C-H···O=C$_{(DKP)}$
		$_{(NH)}$N-H···O=C$_{(DKP)}$ & $_{(Pro-CH)}$C-H···O=C$_{(DKP)}$
		$_{(Pro-CH)}$C-H···C=O$_{(DKP)}$ & $_{(Pro-CH)}$C-H···O=C$_{(DKP)}$
	$D^2_2(6)$	$_{(Pro/DKP-NH)}$N-H···O=C$_{(DKP)}$ & $_{(CH)}$C-H···O=C$_{(DKP)}$
		$_{(DKP-NH)}$N-H···O=C$_{(DKP)}$ & $_{(DKP-CH)}$C-H···O=C$_{(DKP)}$
		[$_{(Pro-CH)}$C-H···O=C$_{(DKP)}$]$_2$
	$D^2_2(7)$	$_{(DKP-NH)}$N-H···O=C$_{(DKP)}$ & $_{(Pro-CH)}$C-H···O=C$_{(DKP)}$
		$_{(DKP-NH)}$N-H···O=C$_{(DKP)}$ & $_{(CH)}$C-H···O=C$_{(DKP)}$
		$_{(Pro-NH)}$N-H···O=C$_{(DKP)}$ & $_{(Pro-CH)}$C-H···O=C$_{(DKP)}$
	$D^2_2(8)$	$_{(DKP-NH)}$N-H···O=C$_{(DKP)}$ & $_{(Pro-CH)}$C-H···O=C$_{(DKP)}$
		$_{(Pro-NH)}$N-H···O=C$_{(DKP)}$ & $_{(DKP-NH)}$N-H···O=C$_{(DKP)}$
		[$_{(Pro-CH)}$C-H···O=C$_{(DKP)}$]$_2$
	$D^2_2(9)$	$_{(DKP-NH)}$N-H···O=C$_{(DKP)}$ & $_{(Pro-CH)}$C-H···O=C$_{(DKP)}$
	$D^2_2(10)$	$_{(Pro-CH)}$C-H···O=C$_{(DKP)}$ & $_{(CH)}$C-H···O=C$_{(DKP)}$
		$_{(Pro-NH)}$N-H···O=C$_{(DKP)}$ & $_{(Pro-CH)}$C-H···O=C$_{(DKP)}$
		$_{(Pro-NH)}$N-H···O=C$_{(DKP)}$ & $_{(CH)}$C-H···O=C$_{(DKP)}$
	$R^1_2(6)$	$_{(Pro-NH)}$N-H···O=C$_{(DKP)}$ & $_{(CH)}$C-H···O=C$_{(DKP)}$
	$R^2_2(8)$	[$_{(DKP-NH)}$N-H···O=C$_{(DKP)}$]$_2$
	$R^2_2(9)$	[$_{(DKP-CH)}$C-H···O=C$_{(DKP)}$]$_2$
	$R^2_2(10)$	[$_{(Pro-CH)}$C-H···O=C$_{(DKP)}$]$_2$
ROPVOH	C(4)	$_{(Pro-CH)}$C-H···O=C$_{(DKP)}$
	C(5)	$_{(Pro-CH)}$C-H···O=C$_{(DKP)}$
	C(6)	$_{(Pro-CH)}$C-H···O=C$_{(DKP)}$
	C(9)	$_{(Pro-CH)}$C-H···O=C$_{(DKP)}$
	$C^1_2(5)$	[$_{(Pro-CH)}$C-H···O=C$_{(DKP)}$]$_2$
	$C^1_2(6)$	[$_{(DKP/Pro-CH)}$C-H···O=C$_{(DKP)}$]$_2$

	$C^2_2(9)$	$_{\text{(Pro-CH)}}$C-H···N & $_{\text{(CH)}}$C-H···O=C$_{\text{(DKP)}}$
	$C^2_2(10)$	$_{\text{(Pro-CH)}}$C-H···C=O$_{\text{(DKP)}}$ & $_{\text{(Pro/DKP-CH)}}$C-H···O=C$_{\text{(DKP)}}$
		$_{\text{(Pro-CH)}}$C-H···N & $_{\text{(Pro-CH)}}$C-H···O=C$_{\text{(DKP)}}$
	$R^1_2(6)$	[$_{\text{(Pro-CH)}}$C-H···O=C$_{\text{(DKP)}}$]$_2$
	$R^1_2(8)$	[$_{\text{(CH)}}$C-H···O=C$_{\text{(DKP)}}$]$_2$
	$R^2_3(10)$	[$_{\text{(DKP-CH)}}$C-H···C=O$_{\text{(DKP)}}$]$_2$ & $_{\text{(Pro-CH)}}$C-H···O=C$_{\text{(DKP)}}$
TUTSOR	D(2)	$_{\text{(CH)}}$C-H···O=C$_{\text{(DKP)}}$
		$_{\text{(OH)}}$O-H···O=C$_{\text{(DKP)}}$
	$D^2_2(8)$	[$_{\text{(CH)}}$C-H···O=C$_{\text{(DKP)}}$]$_2$
		$_{\text{(OH)}}$O-H···O=C$_{\text{(DKP)}}$ & $_{\text{(CH)}}$C-H···O=C$_{\text{(DKP)}}$
		[$_{\text{(OH)}}$O-H···O=C$_{\text{(DKP)}}$]$_2$
	$D^2_2(9)$	$_{\text{(CH)}}$C-H···O-H$_{\text{(OH)}}$ & $_{\text{(OH)}}$O-H···O=C$_{\text{(DKP)}}$
	$R^1_2(7)$	$_{\text{(CH)}}$C-H···C=O$_{\text{(DKP)}}$ & $_{\text{(OH)}}$O-H···O=C$_{\text{(DKP)}}$
UCOBEV	C(6)	$_{\text{(Pro-CH)}}$C-H···O=C$_{\text{(DKP)}}$
	C(9)	$_{\text{(Pro-CH)}}$C-H···O=C$_{\text{(DKP)}}$
	$C^2_2(10)$	$_{\text{(Pro-CH)}}$C-H···O=C$_{\text{(DKP)}}$ & $_{\text{(DKP/Pro-CH)}}$C-H···O$_{\text{(C-O-C)}}$
		$_{\text{(CH)}}$C-H···O=C$_{\text{(DKP)}}$ & $_{\text{(DKP/Pro-CH)}}$C-H···O$_{\text{(C-O-C)}}$
UDALUJ	D(2)	$_{\text{(Pro/DKP-CH)}}$C-H···O$_{\text{(H2O)}}$
	$D^1_2(3)$	[$_{\text{(H2O)}}$O-H···O=C$_{\text{(DKP)}}$]$_2$
	$D^2_2(4)$	$_{\text{(H2O)}}$O-H···O$_{\text{(H2O)}}$ & $_{\text{(H2O)}}$O-H···O=C$_{\text{(DKP)}}$
	$D^2_2(6)$	$_{\text{(DKP/Pro-CH)}}$C-H···O$_{\text{(H2O)}}$ & $_{\text{(H2O)}}$O-H···O=C$_{\text{(DKP)}}$
	$D^2_2(7)$	$_{\text{(DKP/Pro-CH)}}$C-H···C=O$_{\text{(DKP)}}$ & $_{\text{(H2O)}}$O-H···O=C$_{\text{(DKP)}}$
	$D^2_2(8)$	$_{\text{(Pro-CH)}}$C-H···O$_{\text{(H2O)}}$ & $_{\text{(H2O)}}$O-H···O=C$_{\text{(DKP)}}$
		[$_{\text{(H2O)}}$O-H···O=C$_{\text{(DKP)}}$]$_2$
	$D^2_2(10)$	$_{\text{(CH)}}$C-H···O$_{\text{(H2O)}}$ & $_{\text{(H2O)}}$O-H···O=C$_{\text{(DKP)}}$
	$D^2_3(8)$	$_{\text{(DKP-NH)}}$N-H···O=C$_{\text{(DKP)}}$ & [$_{\text{(H2O)}}$O-H···O=C$_{\text{(DKP)}}$]$_2$
		[$_{\text{(H2O)}}$O-H···O=C$_{\text{(DKP)}}$]$_2$ & $_{\text{(DKP-CH)}}$C-H···O=C$_{\text{(DKP)}}$
	C(5)	$_{\text{(DKP-NH)}}$N-H···O=C$_{\text{(DKP)}}$
		$_{\text{(DKP-CH)}}$C-H···O=C$_{\text{(DKP)}}$
	$C^2_2(6)$	$_{\text{(DKP/Pro-CH)}}$C-H···O$_{\text{(H2O)}}$ & $_{\text{(H2O)}}$O-H···O=C$_{\text{(DKP)}}$
	$C^2_2(9)$	[$_{\text{(H2O)}}$O-H···O=C$_{\text{(DKP)}}$]$_2$
	$C^2_2(10)$	$_{\text{(DKP-NH)}}$N-H···O=C$_{\text{(DKP)}}$ & $_{\text{(DKP-CH)}}$C-H···O=C$_{\text{(DKP)}}$
	$R^2_2(8)$	$_{\text{(DKP-NH)}}$N-H···O=C$_{\text{(DKP)}}$ & $_{\text{(DKP-CH)}}$C-H···O=C$_{\text{(DKP)}}$
UKOCON	C(5)	$_{\text{(DKP-CH)}}$C-H···O=C$_{\text{(DKP)}}$
	C(6)	$_{\text{(Pro-CH)}}$C-H···O=C$_{\text{(DKP)}}$
	C(9)	$_{\text{(CH)}}$C-H···O=C$_{\text{(DKP)}}$
	$C^1_2(8)$	$_{\text{(DKP)}}$C-H···O=C$_{\text{(DKP)}}$ & $_{\text{(Pro-CH)}}$C-H···O=C$_{\text{(DKP)}}$
WAZRAQ	C(6)	$_{\text{(DKP-CH)}}$C-H···H-C$_{\text{(CH-Pro)}}$
	C(10)	$_{\text{(DKP-CH)}}$C-H···O$_{\text{(C-O-C)}}$
	$C^2_2(10)$	$_{\text{(Pro-CH)}}$C-H···O=C$_{\text{(DKP)}}$ & $_{\text{(Pro-CH)}}$C-H···O$_{\text{(C-O-C)}}$
		$_{\text{(Pro-CH)}}$C-H···O=C$_{\text{(DKP)}}$ & $_{\text{(CH)}}$C-H···O=C$_{\text{(DKP)}}$
XOLQUL	C(5)	$_{\text{(Pro-CH)}}$C-H···O=C$_{\text{(DKP)}}$
	C(6)	$_{\text{(Pro-CH)}}$C-H···O=C$_{\text{(DKP)}}$
	$C^1_2(4)$	[$_{\text{(Pro)}}$C-H···O=C$_{\text{(DKP)}}$]$_2$
	$C^1_2(5)$	[$_{\text{(Pro)}}$C-H···O=C$_{\text{(DKP)}}$]$_2$
	$C^1_2(6)$	[$_{\text{(CH)}}$C-H···O=C$_{\text{(DKP)}}$]$_2$
	$C^1_2(7)$	[$_{\text{(Pro)}}$C-H···O=C$_{\text{(DKP)}}$]$_2$
	$C^1_2(8)$	[$_{\text{(Pro)}}$C-H···O=C$_{\text{(DKP)}}$]$_2$
	$C^1_2(9)$	[$_{\text{(Pro)}}$C-H···O=C$_{\text{(DKP)}}$]$_2$
	$C^2_2(10)$	[$_{\text{(Pro)}}$C-H···O=C$_{\text{(DKP)}}$]$_2$
	$R^1_2(5)$	[$_{\text{(Pro)}}$C-H···O=C$_{\text{(DKP)}}$]$_2$
	$R^2_2(10)$	[$_{\text{(Pro-CH)}}$C-H···O=C$_{\text{(DKP)}}$]$_2$
YERPIU	C(5)	$_{\text{(DKP-NH)}}$N-H···O=C$_{\text{(DKP)}}$
	C(6)	$_{\text{(Pro-CH)}}$C-H···O=C$_{\text{(DKP)}}$
	C(8)	$_{\text{(Pro-CH)}}$C-H···O=C$_{\text{(DKP)}}$
	$C^1_2(7)$	[$_{\text{(CH)}}$C-H···O=C$_{\text{(DKP)}}$]$_2$
	$C^1_2(8)$	$_{\text{(DKP-CH)}}$C-H···O=C$_{\text{(DKP)}}$ & $_{\text{(Pro-CH)}}$C-H···O=C$_{\text{(DKP)}}$
	$R^2_2(9)$	$_{\text{(DKP-NH)}}$N-H···O=C$_{\text{(DKP)}}$ & $_{\text{(CH)}}$C-H···O=C$_{\text{(DKP)}}$
ZAJZOZ	C(6)	$_{\text{(CH)}}$C-H···O=C$_{\text{(DKP)}}$
	$C^2_2(8)$	$_{\text{(Pro-CH)}}$C-H···O$_{\text{(C=O)}}$ & $_{\text{(Pro-CH)}}$C-H···O=C$_{\text{(DKP)}}$
